# Water pollution generated by tourism: Review of system dynamics models

**DOI:** 10.1016/j.heliyon.2023.e23824

**Published:** 2023-12-20

**Authors:** Martina Pásková, Kamila Štekerová, Marek Zanker, Taiwo Temitope Lasisi, Josef Zelenka

**Affiliations:** Faculty of Informatics and Management, University of Hradec Králové, Czech Republic

**Keywords:** Water pollution, Tourism, Plastics, Microplastics, System dynamics models

## Abstract

This study delves into the intricate dynamics of tourism-induced water pollution through a systematic literature review, aiming to unravel complexities using a system dynamics (SD) modeling approach coupled with the PRISMA analysis methodology. Employing a comprehensive PRISMA analysis of 68 pertinent articles, the study establishes a metamodel for comprehending plastic pollution in water ecosystems resulting from tourism. The methodology emphasizes economic and environmental dimensions, causal conditions, and interventions, with a specific focus on the role of Information and Communication Technology (ICT). The results highlight integrated strategies as crucial in mitigating tourism-induced water pollution. These strategies advocate for the incorporation of environmental conservation and sustainable management practices. The study underlines the pivotal role of environmental education, awareness, and investments in protection as effective interventions. The findings offer valuable insights for policymakers and stakeholders in the tourism industry, emphasizing the necessity for proactive planning and management. The study advocates for knowledge-based decision-making to optimize tourism's environmental impacts and underscores the significance of quick and flexible responses to environmental challenges.

## 1 Introduction

Water is essential to life and water pollution and the introduction of toxic substances to water bodies such as lakes, rivers, oceans, and so on, getting dissolved in them, lying suspended in the water, or depositing on the bed [1], represents one of the most serious ecological threats. The main aim of this study is to analyze the research dedicated to the system dynamics modelling of water ecosystems' pollution generated by tourism. The water ecosystems' pollution in tourism should be researched in a transdisciplinary way with a special focus on the interrelationship with the social responsibility concept and sustainability management (e.g. Ref. [2], sustainability indicators (e.g. carbon footprint – [3], case studies (e.g. Ref. [4], predictive models [5] and ecosystem services (biodiversity economy; [6,7]. As a starting point for the desirable transdisciplinary studies, the intention behind this study is to deliver an analysis of the current situation in the aforementioned research directions.

### 1.1 Water pollution

[8] noted that pollutants are harmful substances that can include organic, inorganic, radioactive materials, and so on. Pollution degrades the quality of water, represents a disaster for aquatic ecosystems, contaminates groundwater sources for household consumption, and indirectly causes water-borne diseases and illnesses. While water pollution can be caused in several ways, industrial waste discharge and city sewage disposals have been noted as the most contributing factors to water pollution [8]. Indirectly, water pollution can be an effect of contamination from groundwater bodies or the atmosphere via rain [8]. Human agricultural practices and improper waste disposal systems are known sources of soil and groundwater pollution [9]. Furthermore, tourism-related marine activities, such as boating, snorkeling, and scuba diving, also contribute to water pollution through the release of oils, fuel residues, and chemicals [10]. The cumulative impact of these activities can degrade water quality, harming marine ecosystems. Research emphasizes the need for sustainable management practices to mitigate these adverse effects [11].

Additionally, the expansion of tourism infrastructure, including the construction of hotels, roads, and ports, can lead to habitat destruction and increased sedimentation in water bodies [12]. Construction activities introduce pollutants such as sediment, heavy metals, and chemicals into aquatic ecosystems, causing long-term damage. Literature underscores the importance of effective environmental impact assessments and sustainable development practices in the planning of tourism infrastructure [13].

Tourists also contribute to water pollution through the improper disposal of solid waste, including plastics, packaging materials, and other non-biodegradable items [14]. The transient nature of tourism exacerbates this issue, as waste management infrastructure may not be adequately equipped to handle the sudden influx of visitors. Studies emphasize the need for awareness campaigns and the implementation of responsible tourism practices to address this aspect of water pollution [15].

The severity of water pollution caused by the tourism industry is evident in the long-lasting ecological consequences observed in many popular tourist destinations [16]. Increased nutrient levels, eutrophication, loss of biodiversity, and disruption of aquatic ecosystems are among the documented impacts [17]. The severity is exacerbated by the cumulative effect of multiple stressors, emphasizing the interconnectedness of various tourism-related activities and their collective impact on water quality.

### 1.2 Impact of tourism

Tourism as a temporary, short-term based on the movement of people to destinations and their temporary stay outside the places where they normally live leads to excessive consumption of single-use plastic items such as food packaging, bottles, or hotel bathroom accessories [3,4].

**Fig. 1:**
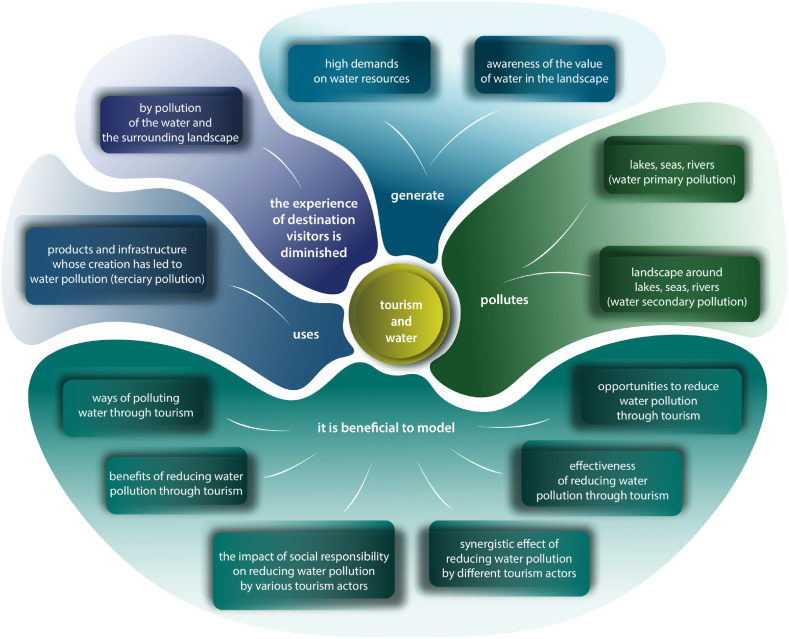
The relationship of tourism to water pollution and water resources. Inspired by [35,36].

Beyond its importance to the existence of the terrestrial life and its criticality in the composition of biosphere, water serves as a crucial source of tourist attraction in coastal and many vitreous destinations [18]. Water ultimately contributes to the experiential quality of tourism activities in water tourism destinations, however, pollution of water and beaches significantly reduces the quality of the experience of water tourism participants [5,19]. The predominant component of visible pollution in oceans with a share of about 80 % (e.g. Ref. [20], is plastics, transmitted to the seas and oceans mainly by rivers [21]. Due to its global rapid growth, transformation, and massification, tourism contributes substantially to the pollution of the environment, especially by plastics [[22], [23], [24], [25]]. On the other hand, tourism itself suffers from this pollution, while visitors prefer clean destinations (e.g. Refs. [5,[26], [27], [28], [29], [30]], and some segments of visitors even virgin/authentic sites (Wang, 1999). According to the social exchange theory [31,32], the deteriorated life quality of local inhabitants increases their tourism irritation, and this results in a decrease in visitors' experience quality [33,34]. The relationship of tourism to water pollution and water resources in the form of a mental map summarizes Fig. 1.

Tourism-led growth hypothesis, which argues that tourism development is an indicator of economic growth and development, has been validated in academic scholarship. Thus, as tourism develops, the host nations also grow economically resulting in infrastructural development and an increased rate of industrialization [37,38]. According to Ref. [39]; the rate of industrialization and economic growth is often measured by the number of plastics in society. He argues that in this optics, the larger the share of plastics, the more developed the economy, and at the same time an increasing amount of plastic waste is destroying the environment. The degree of the problem of plastic pollution is documented by the rapidly increasing production of over 335 million tons of plastics worldwide in 2016 [40] and 400 million tons of plastics worldwide in 2018 [41], buying one million disposable plastic bottles every minute with only 20 % of disposable plastics being recycled since 2015 (United Nations, 2021), and discovering plastic pieces in almost every place on Earth, including Mariana Trench and Mount Everest [42]. Under different conditions of waste management, the development of plastic pollution on Earth until 2060 is modeled [43].

More specifically participation in adventure tourism generates a need for other longer-use plastic outdoor items such as a tent, sleeping bags, rafts/canoes, or surfboards. Both the demand and supply sides of the tourism market are contributing to plastic pollution, directly in destinations and resource regions [44]. applied SD models for single-use plastic reduction initiatives in the food sector in Thailand. The surge in single-use plastics is due to the urgent production of face masks and medical protective equipment during COVID-19 (Nikiema and Asiedu, 2022).

According to WWF (2018), the most popular seaside destination for tourists in the world is the Mediterranean region, which is visited by more than 220 million tourists every year. The organization points out the fact, that during the tourist season, these 220 million people would cause about a 40 % increase in plastic waste in just three months. As stated by Rosian [45]; due to the semi-enclosed position of the Mediterranean Sea and a large number of estuaries such as the Nile, Ebro, Rhone, Po or Ceyhan, and Seyhan in Turkey, this sea is becoming a so-called "plastic trap", and this is the area with one of the highest concentrations of plastic pollution in the world.

According to Ref. [46]; rural tourism's development can lead to pollution of water sources. They noted that commercializing rural areas for tourism purposes results in more visitors coming through, increased infrastructure investments, and changes to land use patterns—all factors that must be carefully managed when expanding rural tourism operations. These factors can contribute to water pollution through increased wastewater production, agricultural runoff, and poor waste management practices. Such pollutants contaminate rivers, lakes, and groundwater supplies, which affects their quality in rural areas [47]. Such pollutants contaminate rivers, lakes, and groundwater supplies, which affects their quality in rural areas [47]. [48] propose redefining rural resources as countryside capital, specifically discussing rural tourism as an example [49]. present evidence for sustainable rural tourism activities to minimize negative environmental impacts, particularly water pollution. Their work contributes to understanding integrated rural tourism as a concept. They assert that integrated rural tourism should take environmental sustainability into account and protect natural resources such as waterbodies to minimize pollution and maintain rural areas' attractiveness.

Urban tourism pollution has also become an increasing problem. Tourism activities, including increased transportation, accommodation facilities, and waste generation, contribute to water pollution in urban areas. Urban water bodies may become more polluted as a result of wastewater discharge, poor waste management techniques, and tourist use of chemical products [50]. [51] highlighted one of the primary drivers behind tourism growth: increasing demand among visitors for new experiences and travel destinations. This demand may lead to increased tourism activities in urban areas, which in turn contributes to water pollution through waste generation, improper disposal methods, and strain on water resources (Mikhailenko et al.). (2020) conducted a literature review on cadmium pollution in tourism environments and found that tourism activities, including hotel wastewater management and increased traffic volumes, contribute significantly to its presence on beaches, coastal waters, and urban parks. However, pollution from these sources can have adverse consequences for tourism destinations [52]. Urbanization itself, which often coincides with urban tourism activities, further compounds water pollution issues [53]. assessed urbanization's impact on river water quality in China's Pearl River Delta Economic Zone and found that urban river waters were significantly more polluted compared to rural rivers. Urbanization leads to an increase in industrial and domestic wastewater discharge as well as pollution release from urban areas, all of which lead to reduced river quality [53].

Tourism and human society with accompanying processes in it can be viewed as complex systems. Therefore, different computer modeling techniques, including models of system dynamics, are applicable. According to Forrester (1961, 1969), system dynamics (SD) aids in understanding the nonlinear dynamics of complex systems over a period of time. Models are developed employing time delays, table functions, internal feedback loops, flows, and stocks. Stock and flow diagrams (SFD) and causal loop diagrams (CLD) are the two primary diagram forms that constitute these artefacts. Typically, CLD captures cardinal system variables and establishes their relationships. Systems Archetypes are universal CLD types that work well in most fields. SFD documents system dynamics and can be applied to a variety of tasks, including scenario evaluation, testing in extreme conditions, sensitivity analysis, boundary testing, and predicting future system behaviour.

## 2 Materials and methods

System dynamics models provide an invaluable means of comprehending complex systems [54]. These mathematical representations of interactions and feedback loops within a system enable researchers to simulate and predict its behavior over time [55]. When applied to tourism-induced water pollution issues, system dynamics models can help researchers simulate plastic waste entering aquatic ecosystems while also assessing different interventions or policies implemented for pollution reduction [56].

PRISMA (Preferred Reporting Items for Systematic Reviews and Meta-Analyses) is an internationally recognized, rigorous approach for conducting systematic reviews [57]. The PRISMA review methodology was employed to thoroughly assess research approaches and results related to modeling tourism-generated water pollution [58]. PRISMA provides a checklist and guidelines to enable transparent and comprehensive reporting of systematic reviews, ensuring all relevant studies are identified, selected, and analyzed impartially and systematically [59]. A recent PRISMA review of scientific contributions published between 2010 and 2022 allowed researchers to synthesize existing research on system dynamics modeling of tourism-generated water pollution [58].

PRISMA analysis was chosen as part of this study due to its transparent and replicable process for conducting systematic reviews [60]. By adhering to PRISMA guidelines, researchers ensured their review was comprehensive, impartial, and followed a rigorous methodology [58]. This methodological approach proved particularly valuable when researching because it allowed for synthesizing existing evidence while simultaneously identifying research gaps or future opportunities [61].

The present study focuses on system dynamics models of the water pollution, generated by tourism. The PRISMA review [62] was conducted. The following research questions were formulated.

Q 1 Which kind of SD models describing sources, transport, and distribution of pollution of water has already been published?
Q 2 Which types of water environments (such as marine, brackish, freshwater, etc.) polluted by tourism-generated debris are frequently researched?
Q 3 What were the models' purposes and temporal scales?
Q 4 What is the geographic distribution of case studies?
Q 5 What system dynamics diagrams and modelling platforms were used? What is the focus of studies on the relationship between tourism water pollution and aquatic ecosystems using SD?

The initial search was undertaken using scientific databases Scopus and Web of Science in February 2023. The review includes full texts published in English, published after 2000. The selection criteria and data-gathering approach centered on system dynamics. in relation to the main topics: water and water ecosystems, pollution, and tourism. Cross-searching was carried out employing the domain-relevant search terms and system dynamics keyword. (Table 1). The keywords and abstracts of articles were examined to exclude papers that failed to satisfy the selected inclusion criteria (Table 2).

**Table 1:** Search terms.

Tourism	Pollution	Water and water ecosystems
Attraction Camp Canoeing Cruise Destination Diving Event Excursion Hospitality Hotel Leisure Rafting Recreation Resort Scenic spot Sea Tourism Tourist Travel Trip Visitor	Contamination Dirty beach Disposal Emission Garbage Litter Plastic Pollutant Pollution Recycling Sewage Toxic Trash Waste Waste Impact	Aquatic Coast Coastal Drinking water Ecosystem Groundwater Lake Marine Ocean River Riverine Sea Smelly water Water Waterfall
Sources: Scopus and Web of Science

**Table 2:** Inclusion criteria.

Criterion	Requirement
Language	English
Type of paper	Journal article, Conference paper, full-text available
Publication	2000–2023
Problem	Exploring the relationship between tourism and pollution of water ecosystems, namely plastic pollution
Methodology	Use of system dynamics modelling to address the problem (consisting of system dynamics equation, causal loop diagram, stock and flow diagram, and/or system archetypes).

**Fig. 2:**
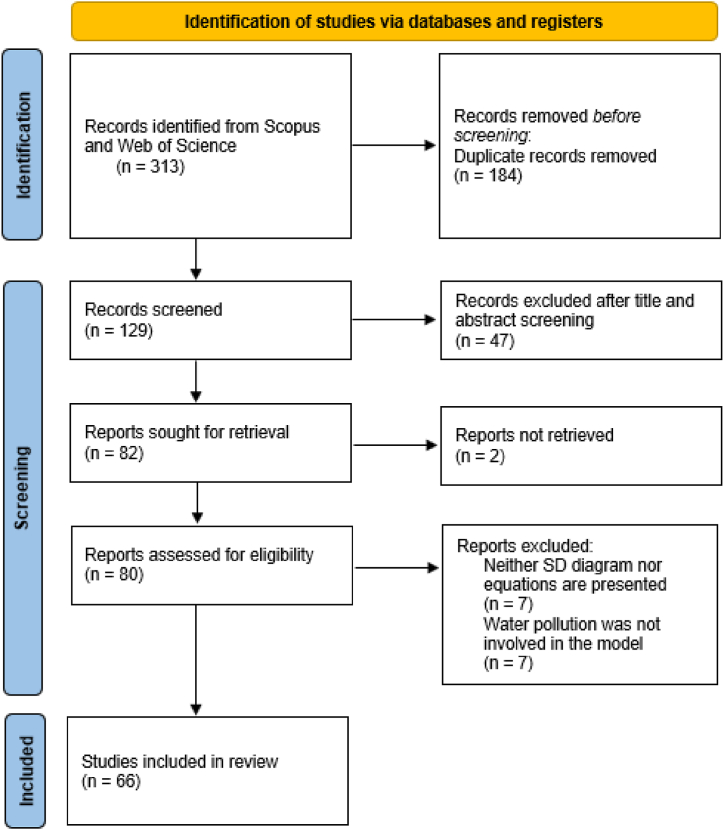
PRISMA flow diagram.

In the first step, 313 results from scientific databases were identified. After removing 184 duplicates, 129 papers were obtained from which 82 papers were sought for retrieval. The rest of the 47 full-text papers were rejected after the title and abstract screening. As two full-texts were not accessible, only 80 papers proceeded to the full-text eligibility assessment stage. Seven papers were excluded in which no system dynamics diagrams or equations were presented, seven other papers were excluded where water pollution was not explicitly captured in the model. Finally, 66 papers (55 journals and ten conference contributions) were analyzed in the frame of both quantitative and qualitative research. Fig. 2 depicts the entire procedure while table 7 (Appendix) presents the selected studies.

Finally, the general scheme (figure 8) situation regarding SD modelling of tourism-induced pollution and degradation of water resources and aquatic ecosystems was derived from the synthesis of the partial results.

## 3 Results

The growing interest in SD modelling in tourism is significant. The majority (91F) of studies were published within the last decade (2012–2023), and over half of them (54 %) between 2018 and 2023. Notably, 2007, 2009, 2012, and 2021 stand out with zero publications, hinting at potential gaps or reduced research activity during those periods. However, a surge in research interest is evident from 2014 onwards, with a peak in 2018. The significant spike in 2018, with 15 papers, might be indicative of a peak in research output, potentially influenced by emerging issues, technological advancements, or increased funding. While certain years, such as 2014, 2015, and 2018, demonstrate a consistent and relatively high number of publications, others like 2007, 2009, and 2012 reveal a lack of research focus during those specific periods. The decline in the number of papers in 2020 and 2021 might be attributed to external factors, such as the global COVID-19 pandemic, which could have disrupted research activities and publication schedules. The overall trend suggests an increasing interest in the topic, especially from 2014 onwards, possibly indicating its growing importance, relevance, or complexity within the academic community.

Interest in SD models grows in general, see e.g. SD review in healthcare [63], transportation [64], and engineering [65].

**Answer to Q1: Which kind of SD models describing sources, transport, and distribution of pollution of water have already been published?**

Three categories of papers with respect to the main topics focused on by authors were identified. A large part of the papers is focused on sustainable tourism and carrying capacity. Usually, authors examine the effects of various potential policies on the ecotourism demand and environmental conditions. These models have been already explored by Ref. [66]. A certain number of models are focused on transport or traffic models, with pollution (of air, water) being one of the important side-effects. Waste production itself, including water pollution by plastics, was, optionally with respect to tourism, presented in the minority of papers.

With respect to tourism itself, the following topics are studied: agritourism [67], cave tourism [68], city tourism [[69], [70], [71]], tourism [[72], [73], [74], [75]], destination image [76], ecotourism, low-carbon, tourism, impact on the ecosystem [[77], [78], [79]], highly aggregated tourist crowds [79], international tourism [80], island tourism/small island tourism [81,82], lagoon ecosystem [83], national park, natural recreation [84], regional tourism, local tourism [85], world heritage [86].

In relation to pollution, the majority of papers discuss waste or pollution in general. Municipal solid waste is a typical type of waste presented in models. Other types of waste are water pollution, solid waste, plastic waste, marine pollution, air pollution, and carbon pollution.

Different types of pollution sources have been studied for water-related ecosystems. Numerous research and various contexts have identified carbon emission as one of the sources of pollution. For instance, a study by Ref. [87] investigated the relationships of five subsystems in Jiuzhai Valley, and carbon emission was one of the parameters taken into account in the environmental representation subsystems and the same context was studied by Ref. [88] in promoting sustainable development. Number of tourists and carbon emissions have been found to be causally related. Using the bottom-up approach to calculate carbon emissions [89], found a causal relationship between transportation behavior and carbon mission in Karimunjawa, Indonesia. In their study of the Barents Sea Region [90], found that among other factors, carbon uptake and export were of interest to the stakeholders. They were concerned about the impacts of climate change on the fishery industry, tour operators, other tourism businesses, environmental, and other non-governmental organizations. In addition [73], discovered a connection between population quality, size, and greenhouse gas emissions in Baoding, China's city [91] conducted a study on the effects of historical and real-world behavior on the endogenous dynamics of the power consumption on the Azorean island of São Miguel. According to the results of their analysis, the island should take into account three crucial system components to accomplish its low-carbon goals: electrification of the transportation sector, increased tourism, and energy efficiency. Similar findings have been drawn from further studies, including [65] in Beijing [83], in the Chiku coastal zone [79], in Xingwen Global Geopark [92], in Galapagos Islands of Ecuador [73], in Dalian city, and [68] in Hinagdanan Cave.

### 3.1 Plastic pollution

Ever since plastic was commercially developed, there has been an accumulating buildup that has resulted in pollution. Human activities result in the production of plastic trash, which is then transported to the ocean and accumulates in the marine ecosystem [93]. Numerous studies have shown that tourism-led growth occurs in Small Island Developing States (SIDS), however [94], in the Maldives found that inorganic wastes and inorganic wastes are harmful to the destination. In Sagarmatha National Park and Buffer Zone in Nepal, environmental degradation is pervasive and is mostly attributed to the uncontrolled expansion of tourism-related activities. Solid wastes (including debris) are no longer the only source of pollution; it is also affecting water quality [95]. Similar research was conducted by Ref. [96] on the management of municipal solid waste (MSW), which includes plastic waste, in touristic islands (Balearic Islands). They found that the main drivers of the MSW generation were the tourist population, resident population, and Gross Domestic Product per capita.

### 3.2 Solid waste and municipal waste

In Sicily [97], asserted that factors influencing tourism demand include the urban environment, transportation infrastructure, natural resources, and cultural resources. In the urban environment, sources of pollution include solid wastes, crowding, and vehicles. Overcrowding, pollution, and water shortages may potentially have an impact on the viability of tourism on Cat Ba Island, according to Ref. [98]. This was also validated in Ref. [99] study of the Cat Ba Biosphere Reserve in Vietnam. The marine ecosystem and coastal environment in Cijin and Kaohsiung, Taiwan, have been significantly degraded by waste from tourism activities [74,75]. The waste and pollution subsystem in Gu et al.'s (2021) study of the Maldives divided solid waste generation into two categories (by locals and tourists), which is similar to Ref. [100]; Luo et al.'s (2020), [85,101]; Pizzitutti et al.'s (2016), and [77] study in Tunisia, Xingwen Global Geopark in China, Tibet, Chiang Mai City, Galapagos Islands of Ecuador, and Rawa Danau respectively The relationship between tourism dynamics and pollution dynamics is found by Ref. [102] as a source of waste loading in Pieh Marine Park, which was validated in Amsterdam [103] and South European island tourist economies [81].

**Answer to Q2. Which types of water environments (such as marine, brackish, freshwater, etc.) polluted by tourism-generated debris are frequently researched?**

With respect to the water ecosystem, most papers discuss the pollution of seas and oceans. Other topics are rivers, canals, groundwater, domestic wastewater, and brackish water.

Twelve publications on water discussed the marine, ocean, and sea, including the following: The study by Ref. [89] focused on Karimunjawa National Park, which is situated in the Java Sea's Karimunjawa Archipelago. While [94] research on the Maldives focused on the management of trash generation, Gu et al.'s (2021) study on tourist recovery post-pandemic in the Maldives took the Indian Ocean into account. Studies on ocean literacy and ocean protection by Refs. [93,104] respectively, focused on the ocean. The Pieh Marine Park in Indonesia served as the core of Nugroho et al.'s (2019) research on the long-term viability of marine protected areas. In a study on the effects of climate change on marine fish [90], took into account ocean warming, acidification, and other environmental factors. The degradation of the marine ecology and coastal environment in Cijin was the focus of the research by Ref. [74]; although with an emphasis on sustainable coastal tourism which was also addressed by Ref. [75]. In their 2018 study, Estay-Ossandon and Mena-Nieto also took into account the Balearic Islands while evaluating the Canary archipelago, one of the most popular tourist destinations in the European Union. In their study on coastal management [105], used the Dutch Wadden Sea as a case study.

There were nine papers on drinking water, freshwater, and domestic water. These include the research conducted by Ref. [69] on sustainable development in a rural area of the Gucheng District of the City of Lijiang. Their findings of this study, which are similar to those of [100] study of Tunisia, showed that as the tourist population rises, drinkable water reduces and low water use may have an impact on locals' quality of life. According to Pizzitutti et al.'s (2017) research on the Galapagos Islands in Ecuador, the expansion of new urban areas is impacted by drinking water, sewage, and electricity. Fresh water supply is one of the sectors that add to the complexity of the tourism industry, according to a study on the sustainability of mass tourism in South European island tourist economies by Ref. [81]. Walsh et al. (2014) studied the well-known dangers to national parks by modeling human dynamics, biocomplexity, and global change. The availability of freshwater was a key consideration for the authors when selecting national parks. In Rawa Danau, Indonesia [77], conducted research on the sustainable management of the freshwater swamp forest as an ecotourism destination.

The rest of the papers focus on less frequent topics such as canals in Amsterdam [103], brackish water in Hinagdanan Cave in the Bohol Island UNESCO Global Geopark [68], or underground water in the Cat Ba Biosphere Reserve in Vietnam [99].

**Answer to Q3: What were the models' purposes and temporal scales?**

The temporal scale of the studies under consideration ranges between 6 months and 5 years, with most models working with a step of 1 year. The details on the temporal scale, the period, and the purpose of each research model are included in Table 3.

**Table 3:** Temporal scale and Purpose of models.

Citation	Temporal scale	Period	Purpose of the model
[80]	1 year	Not specified – 100 years	To analyze the state of underground water
[78]	1 year	2005–2015	Predicate future of ecotourism
[84]	1 year	2008–2030	Analyze tourism in the national park
[104]	5 years	Not specified – 30 years	Increase ocean literacy
[106]	1 month	Not specified – 120 months	Decision Support System
[107]	0,5 year	2007–2017	Secure the sustainability of the wetland
[108]	1 year	2011–2025	Find out the possibility of land use (incl. touristic useable land)
[83]	1 year	2000–2070	To simulate long-term land-use interactions and carbon emissions trends
[93]	1 year	2010–2030	The ocean cleanup project
[109]	1 year	2020–2040	Hotel's adoption of renewable energy technology
[96]	2 year	2000–2030	Show main producers of municipal solid waste
[82]	1 year	1999–2030	Forecasts of the future municipal solid waste generation
[110]	1 month	Jan-17-Jun-20	Tourist behavior research - Tourism recovery strategy
[111]	5 year	2015–2030	Analyze European demand for bio-plastic
[112]	12 months	Not specified - 120 months	Forecast need for human resources in the tourist area
[86]	1 year	2007–2027	Analyze investment into transportation infrastructure for tourism development
[94]	1 year	1979–2050	Analyze waste production
[90]	1 year	2015–2075	Analyze the Barents Sea area
[113]	1 year	1995–2020	Simulates hypotheses of economic growth
[75]	1-time unit	Not specified – 30-time units	Decision Support System for Sustainable Coastal Tourism
[114]	1 year	2000–2018	Analyze the impact of new infrastructure on tourism
[88]	1 year	2000–2100	Analyze the impact of tourism development on eco-environment
[73]	1 year	2001–2028	Analyze ecological system security
[87]	1 year	2013–2025	Analyze the impact of decarbonatization on tourism
[79]	1 year	2010–2030	To measure the carbon footprint
[98]	1 year	2004–2030	Planning for tourism development
[115]	1 year	2006–2015	Decision Support System
[91]	1 year	2005–2050	To analyze the impact of low-carbon law on an isolated island system
[70]	1 year	2015–2055	Study visitors of Cape Town
[71]	1 year	2013–2023	To improve tourism in Surakarta City
[102]	1 year	2003–2040	Interpreting Daly's Sustainability Criteria
[116]	1 day	Not specified – 90 days	Evaluate flooding impacts on municipal solid waste management services
[92]	1 year	2012–2033	Plan scenario for tourism management
[69]	1 year	1990–2050	Minimize the harmful effects of tourism
[117]	1 yar	1930–2022	Analyze total plastic input, microplastic input, and microplastic input to the ocean
[118]	1 year	Not specified – 25 years	To analyze animal ecology and human behavior in Stingray
[119]	1 year	2012–2037	Study the food-supply system in Galapagos
[103]	1 month	Not specified – 120 months	Decision support system
[120]	1 year	2013–2025	To analyze the potential of the destination
[77]	1 year	2020–2030	Management of ecotourism destination
[121]	1 year	2012–2020	To improve cultural heritage sector performance
[89]	1 year	2009–2031	To build a policy scenario for reducing CO2 emission
[74]	1 year	Not specified – 30 years	Decision support system
[122]	1 month	Not specified – 12 months	Integrated plastic management system within an agricultural enterprise
[68]	1 year	2011–2036	To identify the carrying capacity of the cave
[123]	1 year	2009–2030	Sustainable planning
[124]	1 year	2010–2035	Policy modelling for municipal solid waste management
[81]	1 month	Not specified – 720 months	Analyze mass tourism
[125]	1 year	2008–2025	Planning, and optimization of the tourism environment system
[72]	1 year	2014–2054	Show the change in the landscape
[85]	1 year	2000–2050	Development strategies for sustainability

Other studies not presented in Table 3 inclu [67,101]; and [126]; whose purpose of the model is the decision support system [97,100]. analyzed the tourism sector in Tunisia, and Sicily respectively, while [127] evaluated natural destinations and their visitors. The purpose of [76] research model is to understand the complexity of Ethiopia's image as a tourism destination, while for [128]; to improve tourism in Slovenia. Furthermore, developing social-ecological system indicators was the aim of [105] model, for [99] identified sustainability leverage points, and [129] examined the condition of the endangered animals. The focus of [130] model is to reduce the amount of plastic pollution in the ocean in Indonesia [95], focus was on creating a waste plan, and Walsh and Mena's (2014) study model was aimed at analysing the threats to the national park.

One of the purposes of the model is to study the safety of overcrowded areas which was conducted by Ref. [131]; carrying out a thorough investigation of the accidents involving densely populated tourist crowds that also identified the occurrence mechanism and mitigation strategies. Other purposes include.

1. Analysis of the state of the environment/ecosystem in relation to sustainable tourism as seen in Ref. [80]; where the authors applied the Amtoudi Oasis in Southern Morocco, Northern Sahar. A similar purpose was found in Ref. [78] study of Taleqan County in Alborz province, Iran.
2. Prediction: some of the articles such as [82,112] aimed at forecasting the need for recourses in agritourism and future municipal solid waste generation respectively.
3. Decision support and planning was another purpose identified in studies such as [85,131]; and [128]. [85] dynamically assessed future sustainability and compared the evolution of sustainability from 2014 to 2050 under various development strategies [131]. study also aimed at providing a high-quality management response for safety precautions for highly aggregated tourist crowds [128]. study also aimed at understanding how the Slovenian Tourism development plan and policies should be systematized and enhanced to enable more comprehensive innovation management.
4. Analysis of tourism in specific destinations towards improving destination management was found to be the motivation in studies such as [100,110]; and [127].
5 [104]. study was geared toward educational purposes by increasing ocean literacy.
6. Simulate long-term period in relation to process: land use interactions and carbon emissions (e.g. [83],

Temporal scale:

The temporal scale of the model was not presented in 15 papers.

The Time step is one year in 49 papers, while 6 papers’ time step is 1 month [116] simulated the period of 90 days (the shortest period among all models), and [122] operated with 12 month period. The longest period: 110 years from 1990 to 2100 was studied in Ref. [69]. Typically, simulation periods start between 2005 and 2015 (close to the date of publishing the paper) and simulations take tens of years steps, e.g. papers attempt to predict the future, e.g. the period 2008–2027 in Ref. [86]; 2012–2037 in Ref. [119] or 2014–2050 in Ref. [85].

**Answer to Q4: What is the geographic distribution of case studies?**

Most models focus on particular destinations from all over the world, e.g., Brazil [80], Iran [78], Thailand [101], South Korea [72], Tibet [85], Nepal [95], Norway [90], Mexico [95]. There are also studies describing models of small island destinations, attractive to international visitors such as the Canary Islands [82], the Cayman Islands [118], Maledives [94,110] and Galapágos [119].

China and Taiwan locations are analyzed in 17 papers, followed by Indonesia (9 papers). Case studies from multiple locations were provided by Walsh et al. (2014), and the global ocean was studied by Ref. [93]. Location was not specified in the five papers.

**Answer to Q5: What system dynamics diagrams and modeling platforms were used?**

The distribution of the modelling platform reveals interesting insights into the preferences and trends within the field. Vensim emerges as the most prominently used software, constituting 46 % (23 papers). This dominance suggests a strong preference or perhaps a high level of functionality and user-friendliness associated with Vensim among researchers or practitioners in System Dynamics [132]. Following Vensim, Stella accounts for 18 % of the usage, indicating a notable but comparatively smaller share. Stella, known for its user-friendly interface and graphical modeling capabilities [133], seems to be a popular choice, albeit to a lesser extent than Vensim. Powersim also holds a substantial share, representing 16 % of the reported software usage. Powersim is recognized for its simulation and modeling capabilities [134], and its presence in a significant portion of the cases underscores its relevance in the System Dynamics modeling landscape. The "Not Specified, Own" category, encompassing 16 % of the cases, introduces an interesting dimension. This may imply that a notable proportion of researchers or modelers either use proprietary or customized software solutions tailored to their specific needs. The lack of specification may also indicate a diverse range of tools used by different individuals or groups within the System Dynamics community. MapSys and Simulink each contribute a modest 2 % to the overall distribution. MapSys, although less commonly used, might have niche applications within certain contexts, while Simulink, a powerful tool for model-based design [135], appears to have a relatively smaller footprint in the creation of System Dynamics models compared to other software options. More than one modelling platform was used by Refs. [98,103,107]; and [89].

Additionally, Causal loop diagrams (CLD) only were presented in 16 papers. CLD and archetypes were presented in two papers. Stock and flow diagrams (SFD) only were presented in 19 papers. CLD and SFD were presented in 34 papers.

**Answer to Q6: What is the focus of studies on the relationship between tourism and water pollution and aquatic ecosystems using SD?**

### 3.3 Focus of studies

[96] considered how tourism contributes to waste production. Municipal solid waste generation in the Balearic Islands is investigated. The production of solid waste by tourists and locals until 2030 is forecasted. Similarly, [94]; explored environmental pollution in the Maldives with respect to the number of tourists per year until 2050 [85]. investigated sustainable tourism in Tibet under several scenarios up to 2050 using CLD and SFD. The simulation's outcomes include tourism enterprise value, tourist-related employment, number of tourists, and pollution. Using CLD and systems archetypes like shifting the burden (international aid), the tragedy of the commons (carrying capacities in tourism), and fixes that fail (tourism development) [99]. identified key sustainability factors in the tourist area of Cat Ba Biosphere Reserve, Vietnam.

[81] focused on mass tourism sustainability in island economies. A complex SFD was created by the authors aimed at accommodation capacities, waste, energy and water supply, visitor numbers, and transport. The simulation provided predictions for the requirement for accommodation capacities, tourism impact on price, and the total number of tourists for 720 months under various scenarios.

In collaboration with local organizations [123], sought to develop Bali's touristic villages sustainably. The simulation's results included the projection of sacred places, green space, settlements, and areas of paddy fields until 2030 under several scenarios. In their case study of Pieh Marine Park [102], focused on the marine protected areas' sustainability. Their initial SFD captured pollution, non-renewable resources, and renewable resources, while their CLD demonstrated a connection between the key elements of the marine park (coral reef condition, pollution, visitor numbers, and fish population). The primary SFD linked the marine park's key variables. The simulation was created to forecast pollution, fish, and coral populations up to 2040 under various scenarios. Similarly, using a sustainable fisheries model and a tourist model, the socioecological system in the Dutch Wadden Sea region was investigated by Ref. [105]. The touristic sub-model included variables that measured sustainability, investment in tourism, proportions of flora and fauna, visitor number, and satisfaction. Only a few studies explored tourism generally; for instance Ref. [73], examined ecological system security in the case study of Dalian, China's coastline tourist city. CLD demonstrated links between tourism-related variables, the environment, and economics. SFD focuses mainly on population size, visitor numbers, and GDP. The simulation predicts the marine population, tourism income, and number of visitors until 2028 under three possible scenarios. Other articles examined coastal tourism. In their 2018 study, You et al. focused on South Korean coastal regions' changing landscapes. Coastal forests, coastal grassland, and coastal sand dunes were shown to vary in relation to tourism infrastructure up to 2054 using SFD. The authors created two distinct scenarios, the first of which was centered on the value of ecosystem services and land erosion. The second scenario was updated to assess how the ecosystem services are impacted by the landscape plan. Several studies examined how tourism contributes to waste production [96]. conducted research on the generation of municipal solid waste in tourist islands using a case study of the Balearic Islands. According to several scenarios, the research estimated that visitors and locals will generate solid waste up to the year 2030.

Using the Maldives as a case study [94], explored waste production. The primary factors in SFD's analysis of environmental pollution and economic growth were the tourism supply and demand, amount of waste, and number of visitors. The waste sub-model was also thoroughly processed and the simulation provided annual predictions for waste, revenue, and visitors up until 2050 under different scenarios [68]. applied SD modelling to identify a sustainable carrying capacity of the cave system in the Philippines, with an interesting ambition to develop a model archetype that “*can also be tailored-fit to address the uniqueness of characteristics and attributes of any tourism system*”. In relation to water, the authors mentioned “*water-related results from human activities”* such as *“alteration of water chemistry, alteration of* cave *hydrology and introduction of alien materials such as pollutants, nutrients, animal species, algae, and fungi.”*

Recent work focuses on the challenges posed by the COVID-19 pandemic and its negative impact on tourism (hand in hand with the positive effect on the natural environment). While [94] addressed the problem of tourism growth and related waste generation in Maldives [110], examined the tourism recovery strategies for the same destination. Small exotic islands are devastated by tourists, but nowadays their economies suffer from the lack of visitors [105]. adopted a group model-building approach *as a diagnostic participative tool for understanding the determinants of characteristic* social-ecological systems *(SES).*

In some papers, tourism is not involved in models explicitly. For example, the Shanghai municipal solid waste model [124] operates with permanent residents and migration residents, but tourism as a phenomenon is not discussed. The limitation of SD models lies in insufficient empirical data; e.g. Ref. [110], compare four new tourism strategies (*social distancing, tax reduction strategy, travel bubble strategy, joint strategy*) which are so new that data are not available.

#### 3.3.1 Variables in models

In Table 4, various models explore the relationship between tourist-related variables, water-related variables, and pollution-related variables [121]. investigate tourists, tourists' satisfaction, and tourists' needs without delving into water or pollution factors [105]. consider the use value for tourists, the number of tourists, and spending per tourist, incorporating mussels and the degree of sustainability in tourist facilities [69]. focuses on tourists, tourism business owners, and tourism services, with an emphasis on water consumption and water quality. Walsh et al. (2014) distinguish domestic tourists, foreign tourists, and tourists in Galapagos, examining boat-based domestic tourists and tourists in Galapagos but excluding pollution-related variables [70]. assesses tourists' coming and leaving rates, omitting water or pollution considerations [102]. examine the number of tourists and tourist amenities, correlating them with fish population, coral reef coverage, and pollution-related variables such as water quality, waste, waste treatment, waste discharge rate of tourists, and fraction of waste polluting the environment [103]. explore tourism area, tourist attractions, tourists per year, tourist revenues, and tourist investments, integrating canal waste treatment and environmental state, pollution, and waste treatment [89]. consider the number of tourist subsystems, the number of domestic tourists, and the number of foreign tourists, linking them to CO_2_ emissions from the ferry, CO_2_ emission subsystems, total CO_2_ emission from mini tour buses, and total CO_2_ emission from private cars [67]. analyzes tourists' flow, mass tourism, and tourism infrastructure without explicit water-related or pollution variables, though environmental degradation factors are included.

**Table 4:** Models with No. of tourists.

Citation	Tourists related variables	Water-related variables	Pollution related variables
[121]	Tourists, Tourists satisfaction, Tourists' needs	Not mentioned	Not mentioned
[105]	Use value for tourists, Number of tourists, Spending per tourist	Mussels	Degree of sustainability in tourist facilities
[69]	Tourists, Tourism Business Owners, Tourism Services	Water Consumption, Water Quality	Not mentioned
[136]	Domestic tourists, Foreign tourists, Tourists in Galapagos	Boat-based domestic tourists, Tourists in Galapagos	Not mentioned
[70]	Tourists coming rate, Tourists Tourists Leaving rate	Not mentioned	Not mentioned
[102]	Number of tourists, Tourist amenities	Fish population, Coral reef coverage	Pollution, Water Quality, Waste, Waste treatment, waste discharge rate of tourists, fraction of waste polluting environment
[103]	Tourism Area, Tourist attraction, Tourists per year, Tourist Revenues, Tourists Investments	Canal Waste Treatment	Environmental State, Pollution, Waste Treat
[89]	The number of tourist subsystem, The number of domestic tourists, The number of foreign tourists	The CO2 emission from the ferry	The CO2 emission subsystem, Total CO2 emission from mini tour bus, Total CO2 emission from private car
[67]	Tourists Flow, Mass Tourism, Tourism Infrastructure	Not mentioned	Environment, Environmental degradation Factor

Table 5 highlights models where plastic waste is seldom represented in model variables [93]. focus on plastic waste in streams and oceans, initial plastic waste in the ocean, and target plastic waste levels, while [130] address plastic bag usage bans, plastic waste, and plastic waste piles at landfills.

**Table 5:** Models with plastic waste.

Citation	Tourists related variables	Water-related variables	Pollution related variables
[93]	Not mentioned	Plastic waste in streams, Plastic waste in the ocean, Initial plastic waste in the ocean, Target plastic waste level in the ocean	Waste generation, Waste generation rate, Plastic waste generation, Plastic waste littered
[130]	Not mentioned	Not mentioned	Plastic Bag Usage Ban, Plastic Waste, Plastic Waste Piles at Landfill, etc.

Table 6, models frequently aim to identify feedback loops in various contexts [101]. examine the number of tourists, domestic tourists, international tourists, and total attractiveness in connection with the attractiveness of wastewater disposal and wastewater [100]. consider the number of tourists and tourism investments in relation to wastewater, pollution, and waste generation [127]. assess strong purist visitors, attractiveness of the site, moderate purist visitors, neutralist visitors, and non-purist visitors without explicitly mentioning water or pollution variables [129]. explore the number of tourists, hotels and restaurants, tourism revenue, attraction of CB islands, and tourism service, without incorporating water or pollution considerations [97]. investigates the number of tourists and the attractiveness of Sicily, connecting them to the attractiveness of Sicily itself and pollution-related variables like solid waste [128]. center on tourist destination development, sustainable, and spatial development without explicit water-related variables [131]. examine the pressure of tourist gatherings, the stimulation of attractive elements, the environmental pressure of traveling, and the psychological status of tourists, without explicitly considering water or pollution factors.

**Table 6:** Models aim to identify feedback loops.

Citation	Tourists related variables	Water-related variables	Pollution related variables
[101]	Number of Tourists, Domestic Tourist, International Tourist, Total Attractiveness	The attractiveness of Wastewater Disposal	Wastewater, Wastewater disposal
[100]	Number of tourists, Tourism investment	Wastewater	Pollution and waste generation, Solid waste Wastewater
[127]	Strong Purist visitors, Attractiveness of the site, Moderate Purist visitors, Neutralist visitors, Non-purist visitors	Not mentioned	Not mentioned
[129]	Number of Tourists, Hotels & restaurants, Tourism revenue, Attraction of CB islands, Tourism Service	Not mentioned	Not mentioned
[97]	Number of Tourists, Attractiveness of Sicily	Attractiveness of Sicily	Solid waste, Pollution
[128]	Tourist destination development	Not mentioned	Sustainable and spatial development
[131]	The pressure of tourist gatherings, Stimulation of attractive elements, Environmental pressure of traveling, The psychological status of tourists	Not mentioned	Not mentioned

## 4 Discussion and summary

**Fig. 3:**
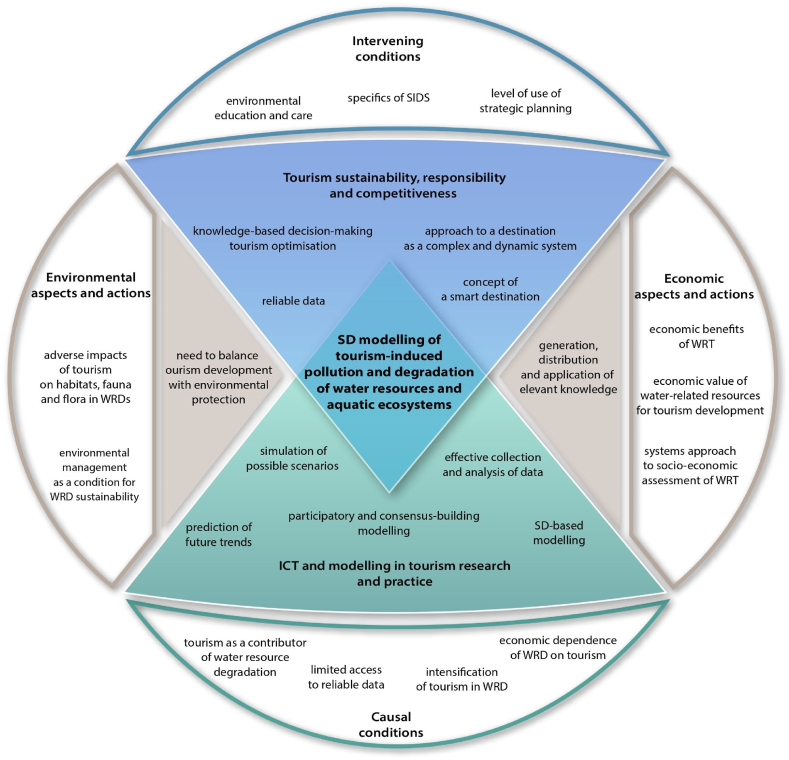
General scheme describing the situation regarding SD modelling of tourism-induced pollution and degradation of water resources and aquatic eco-systems.

Research released the general situation regarding SD modelling of tourism-induced pollution and degradation of water resources and aquatic ecosystems, which is illustrated by a general scheme (Fig. 3). The need to balance tourism development with environmental protection was identified as the main drawing force while creating, disseminating, and using relevant knowledge as a relevant approach for both research and practice. The ICT and modelling have been implemented in tourism research and practice with the aim of achieving sustainability, responsibility, and competitiveness in water-related tourism destinations. Both economic and environmental aspects and actions are described as well as both causal and intervening conditions.

### 4.1 ICT and modelling in tourism research and practice

SD is an effective strategy for addressing environmental concerns related to tourism. SD emphasizes integrating economic, social, and environmental elements for long-term sustainability; many studies have explored its application in studies related to water pollution related to tourism. SD-based modeling has become an effective means of understanding the causes of waste generation in tourism destinations. By identifying key contributing factors, including tourist activities, infrastructure development and management practices, and waste disposal policies, these models can assist with developing strategies to minimize waste production and limit water pollution [137].

Effective collection and analysis of data related to tourism-related pollution of water resources and aquatic ecosystems are integral to informed decision-making. Tools and techniques, such as water quality monitoring systems and data analysis methods, can offer invaluable insight into the sources and impacts of pollution; using this data, targeted interventions to mitigate it can then be developed [138]. SD modeling can aid decision-making and policy development for solid waste and water quality management in environmentally sensitive tourism destinations.

By simulating various scenarios, policymakers can analyze the potential impacts of tourism activities on water quality while identifying effective measures to decrease pollution. SD modeling also assists resource allocation while encouraging sustainable management practices [139]. Simulation scenarios are powerful tools for identifying and assessing solutions and measures related to tourism's impacts on water quality and management. By simulating various scenarios, policymakers can assess the efficacy of various interventions as well as identify the most suitable strategies to counter water pollution; this enables informed decision-making and proactive resource management [139].

Proactive planning and management require tools that allow us to predict future trends related to tourism's contribution to water pollution. By employing predictive models and forecasting techniques, policymakers can anticipate the potential impacts of tourism growth on water resources, giving policymakers insight into adaptive mechanisms and strategies needed to minimize water pollution while supporting sustainable tourism practices [78].

### 4.2 Tourism sustainability, responsibility, and competitiveness

Knowledge-based decision-making is essential to optimizing tourism's environmental impacts. Research shows that residents' support for tourism development depends heavily on their perceptions and concerns regarding its impacts [140]. Policymakers can then make more informed decisions that minimize negative environmental effects while simultaneously maximizing benefits [141]. Reliable data is essential to effective decision-making in tourism-related environmental studies. Studies have emphasized the significance of collecting and analyzing tourism-related pollution of water resources and aquatic ecosystems, offering insights into the sources and impacts of pollution that enable policymakers to formulate targeted strategies for water quality management [142].

Optimizing destination resource allocation is critical to sustainable tourism development. Utilizing technology and data for resource optimization is an integral component of smart destinations, contributing to reduced environmental impacts of tourism [143] while simultaneously mitigating waste generation and water pollution [144]. Tourism requires quick and flexible responses to environmental challenges. Being responsive and adaptable to changing environmental conditions is key to mitigating tourism's negative impacts on water resources, according to studies [145]. By taking timely steps, destinations can prevent and mitigate water pollution issues.

New visitor management options may also help minimize water pollution. Studies have investigated innovative strategies, like community-based tourism and cultural tourism, that engage visitors while simultaneously encouraging sustainable practices [146]. Engaging visitors in environmental conservation efforts allows destinations to reduce the negative impact on water resources. An approach that improves water-related ecosystems as complex systems with nonlinear behaviors is vital for understanding and controlling pollution in tourism destinations. Studies have highlighted the need for comprehensive environmental impact assessments that consider ecological, social, and economic considerations [147]. By adopting such a holistic strategy, policymakers can devise solutions that address complex interactions and feedback mechanisms related to pollution issues in tourism destinations.

### 4.3 Economic aspects/actions

Studies have clearly illustrated the negative consequences of natural resource degradation on the economic competitiveness and attractiveness of the tourism industry growth [148]. Degradation can negatively impact tourism industry growth as well as overall attractiveness [149]. Water pollution may result in declining quality that deters tourists and ultimately impacts economic viability. Water tourism plays an essential role in creating income and employment. Studies have highlighted its significant economic contributions, particularly at coastal and island destinations [150]. Accessible resources and the attractiveness of destinations that feature water are major influences that drive demand and produce economic benefits for communities [99].

The water-related ecosystem is an integral element of tourism services and destinations that rely heavily on aquatic environments, with quality and availability directly impacting tourist experiences and satisfaction levels [151]. Studies have highlighted the significance of maintaining clean and abundant water sources to maintain sustainability and competitiveness for tourism destinations dependent on aquatic features [152]. An integrated systems approach to assessing the socio-economic effects of water tourism can provide invaluable information for destination management. By considering the complex and dynamic nature of these destinations, such an assessment provides a thorough understanding of their interdependencies and feedback mechanisms, which in turn affect tourism's socio-economic impacts [153]. 10.13039/100014337Furthermore, such an approach provides crucial support in decision-making and policy-creation processes to ensure sustainable management [154].

### 4.4 Environmental aspects/actions

Tourism-induced alteration of habitats found in water-related ecosystems is a pressing environmental concern. Tourism activities expanding into coastal areas, wetlands, coral reefs, and other sensitive ecosystems may lead to habitat degradation and loss [155]. Studies have revealed how infrastructure development, pollution from tourism activities, and physical disturbances due to tourism activities can have adverse impacts on these habitats, altering biodiversity and biocomplexity [155]. Tourism's impacts on water resources and aquatic ecosystems have long been documented, from solid waste generation and trash accumulation to degraded water quality [156]. Studies have highlighted the significance of effective waste management practices to mitigate any negative consequences tourism activities may have on these environments [157].

Changes to water chemistry caused by tourism can also have serious repercussions, with the discharge of untreated wastewater, the use of chemicals in tourism-related activities, and the introduction of invasive species all having detrimental impacts on aquatic ecosystems [158]. Tourism-induced threats to biodiversity and biocomplexity in water-related ecosystems are becoming an increasing source of concern. Human presence, habitat alteration, and pollution associated with tourism activities may disrupt ecosystems and threaten species' survival [155]. Studies have noted the need for conservation efforts and sustainable management practices that preserve this vital natural resource [159].

Ecological security is of critical importance in tourism destinations for their long-term viability and the sustainability of eco-socioeconomic systems. It encompasses protecting natural resources such as water bodies for long-term tourism activities [73]. Studies have highlighted the significance of including ecological security principles in tourism policies and management strategies to foster sustainable development [160]. Water and waste management are essential elements of the sustainability of tourism destinations. Effective water management practices include conservation, wastewater treatment, and sustainable use of resources [161]. At the same time, proper waste management must also take place to prevent pollution of these waters [157].

### 4.5 Causal conditions

Tourism is an influential source of water pollution and resource degradation [162]. Tourism activity in destinations has increased the production of waste such as sewage, solid waste, and chemical pollutants [156], which in turn have adverse impacts on water quality, ecosystems, and biodiversity [163], as well as on coastal regions particularly susceptible to impacts of pollution [164]. One of the greatest challenges associated with water pollution is access to accurate data [163]. Accurate data about its sources and impacts is essential for effective management and mitigation strategies, yet data collection efforts often fall short, especially in developing nations [164]. Without sufficient information available to assess its scope and devise targeted interventions,

Studies conducted previously have highlighted the detrimental environmental impacts of tourism on water resources. One such research effort in China revealed that tourism activities led to an increase in water pollution at West Lake Basin due to an increase in tourist numbers and economic income associated with tourism [162]. A further investigation in Romania demonstrated a direct and significant relationship between tourist activities and environmental degradation and their subsequent degradation, emphasizing the necessity of sustainable tourism practices [165].

Asserting measures against pollution and degradation of water resources at tourism destinations requires taking an integrated approach. Environmental conservation and sustainable management practices should be prioritized [166]. This should include implementing efficient waste management systems, encouraging responsible tourism practices, and raising awareness among tourists and local communities regarding water resource conservation [165]. Furthermore, policymakers should enact policies and regulations that incentivize sustainable tourism practices while discouraging harmful activities [167].

### 4.6 Intervening conditions

Environmental education and awareness play an essential role in fostering sustainable practices and mitigating the negative impacts of tourism on water-related ecosystems. Previous studies have illustrated its importance for changing tourists' behaviors and inculcating responsible environmental practices [168], with situational environmental education having positive influences on behavioral intentions as well as responsible environmental behavior [169]. Therefore, including environmental education initiatives in water tourism practices could significantly contribute to raising awareness while encouraging sustainable tourism practices.

Environmental investments are essential in mitigating the negative environmental impacts associated with tourism pollution. Research has indicated that destination environmental attributes play an important role in shaping perceptions [170]. Environmental protection investments can enhance a destination's image and draw in tourists who prioritize sustainability. Studies have also highlighted the necessity for sustainable tourism development in small island developing states (SIDS) [171,172]. SIDS face unique challenges due to their vulnerability to climate change and limited resources [173]. Therefore, investments in environmental protection for SIDS are imperative for maintaining their unique ecosystems while guaranteeing tourism's long-term sustainability.

Strategic planning plays a critical role in controlling the intensification of tourism-induced water pollution, helping anticipate and address its potential negative effects on water resources. Unfortunately, however, research on the use of strategic planning in tourism pollution remains limited compared to its application in other fields. One of the few studies that have been on pro-environmental behavior often uses quantitative approaches such as structural equations or regression analysis [174], suggesting more comprehensive investigations on its application in managing intensified tourism-induced water pollution.

The use of systematic literature review in the tourism and hospitality field is gaining momentum as seen in studies such as [175] where the authors carried out a systemic review of systemic reviews in tourism. They found that multiple systematic reviews did not clearly explain their data-gathering process, which caused a lack of clarity in the data collection and study results. They suggested that future systematic reviews might be based on more reliable and transparent standards, which are essential to reducing implicit bias and researchers' prejudice, which was taken into consideration in thisstudy. Other tourism areas that this methodology has been used include augmented and virtual reality [176], disaster and climate change [177,178], ICT in sustainable tourism [179], and water quality indices [180].

## 5 Conclusions

A review of SD in tourism has already been presented by Ref. [66] that demonstrated the effectiveness of system dynamics models for planning and making decisions in the tourism industry, identifying externalities driven by tourism, and forecasting both its positive and negative effects. Based on their study, system dynamic models in tourism-generated water pollution studies has been reviewed. The focus on SD is because when studying an ecosystem, it is important to analyze non-linear interactions and processes on a large scale and with their long-term impacts. These processes can be well captured by SD models which provide a new perspective. Although it is obvious that tourism contributes significantly to the plastic pollution of (not only water) ecosystems, it still has not been explored deeply using SD models. System dynamics models are either focused on pollution of the environment or tourism itself, but rarely both. Here a research gap of less deeply and systematically studied pollution processes has been identified, as such this study used a metamodel of plastic pollution in the water ecosystem caused by tourism activities using 68 related articles to proffer answers to all the research questions.

The result of thereview indicates that carrying capacity and sustainable tourism are major topics of discussion in the papers. Typically, authors examined the effects of various political actions on the state of the environment and the demand for ecotourism. Air and water pollution are significant side effects in a number of models that are centered on transportation or traffic simulations. A small number of publications described waste generation as a whole, including plastic pollution of water as it relates to tourism. The majority of studies discussed ocean and sea pollution in relation to aquatic ecology. Rivers, canals, groundwater, household wastewater, and brackish water are other topics. The research under consideration spans a variety of periods, including 1-time unit, 6 months, and 5 years. Analysis of the state of the environment and ecosystem in relation to sustainable tourism, forecasting, decision planning and support, better destination management, education, and simulation of long-term periods in relation to process are some of the objectives of the models. Furthermore, China and Taiwan locations are the geographical locations that were mostly analyzed, followed by Indonesia, and Vensim, Stella, and Powersim are the most popular modelling platform used. Three variables were identified as the focus of the studies’ model: the number of tourists/visitors, plastic waste, and identified feedback loops.

The contribution of the study is in three folds. Firstly, the importance of ICT in tourism research modelling has been identified, particularly, system dynamics, which is a tool for the effective collection and analysis of data associated with tourism-related pollution of water resources and aquatic ecosystems. This will support decision decision-making and policy development for solid waste and water quality management in environmentally sensitive tourism destinations, simulation scenarios as a tool for identifying and evaluating solutions and measures related to tourism impacts on water quality and water management. Secondly, the study's findings emphasize the importance of knowledge-based decision-making to optimize the environmental impact of tourism in increasing the destination's ability to optimally allocate resources and ensure flexible and quick responses to environmental challenges to achieve tourism sustainability and competitiveness. Lastly, environmental education and awareness in water-related destinations as well as investments in environmental protection in water-related destinations are identified as conditions that can intervene in tourism water-related pollution. Findings of this study will support future study directions by assisting scholars and decision-makers in understanding trends and developments in the water pollution impact of the tourism industry.

It is recommended that future studies should accommodate other methodologies to further understand the impact of tourism on the water ecosystem. Other analytical methods such as the Theory-Context-Characteristics-Methods (TCCM) create room for exploring the uncovered or less attended areas and develop theoretical models from the perspective of less explored countries to be able to generalize the research in the subject domain. Future research can take into account the interaction of social and cultural aspects because it is still challenging to fully understand the natural ecosystem, human adaptability, and the impact of their connection with nature. Further exploration and refinement of system dynamics models can provide a better understanding of the complex dynamics of pollution in water ecosystems resulting from tourism activities. These models can be enhanced by incorporating variables such as waste management practices, tourism growth patterns, and the influence of socioeconomic factors. Finally, establishing long-term monitoring programs to assess the effectiveness of pollution mitigation measures and policies, while continuously evaluating the state of water ecosystems in tourism destinations, can inform adaptive management strategies and ensure the long-term sustainability of these environments. By addressing these research directions, the understanding of tourism-related pollution can be advanced, effective mitigation strategies, and promote sustainable practices in the tourism industry to protect and preserve water ecosystems.

### 5.1 Limitation and future research direction

Though this study provides invaluable insight into tourism-induced water pollution, several limitations should be kept in mind. First, its focus on system dynamics modeling may exclude other relevant approaches like agent-based or mathematical modelling that could shed additional light on this complex issue. Future research should compare and contrast various modeling techniques to gain a fuller understanding of this complex topic. Second, restricting itself solely to English-language publications may create a language bias and miss important insights from non-English literature. Tourism being an international phenomenon, research from diverse linguistic backgrounds may enrich the understanding of diverse cultural and environmental contexts; future studies could utilize multilingual research teams or translation services to fill this linguistic void.

One limitation lies in the publication date range, primarily covering papers published from 2000 to 2022. While this timeframe captures recent developments, it may miss historical research that could offer context and long-term trends related to tourism-induced water pollution. Future studies should conduct retrospective analyses in order to incorporate previous studies. Plastic pollution may overshadow other pollutants such as chemical contaminants, nutrient runoff, and sedimentation that also have negative impacts on aquatic ecosystems. Future studies must aim for a more comprehensive examination of all the pollutants associated with tourism activities.

To overcome these limitations and increase knowledge of tourism-induced water pollution, future research avenues should be explored. Adopting an interdisciplinary approach that incorporates various modeling techniques—system dynamics, agent-based modeling, and mathematical modeling—may give researchers a more in-depth view of its complexity. Using different modeling approaches allows researchers to capture different aspects of an issue, which enables more robust policy recommendations. Beyond pollution and system dynamics, several promising avenues should be investigated. An essential direction would be examining how climate change contributes to tourism-induced water pollution.

Climate change impacts, such as altered precipitation patterns, rising temperatures, and sea-level rise, can exacerbate pollution dynamics in tourist destinations. Future research should investigate the interactions between climate change and tourism activities, specifically how changing weather conditions and extreme events could contribute to increased pollution incidents that negatively affect water ecosystems. By including spatial perspectives in future research, incorporating a spatial dimension may also deepen understanding of tourism-induced water pollution. Geospatial analysis and Geographic Information Systems (GIS) can be invaluable tools for mapping pollution hotspots, identifying vulnerable areas, and assessing tourism-related impacts on a geographical scale. By adopting this spatial perspective, researchers can offer targeted recommendations for managing pollution at particular destinations.

## Funding

The financial support of the Specific Research Project Information and Knowledge Management and Cognitive Science in Tourism of FIM UHK is gratefully acknowledged.

## Data availability statement

The data supporting the findings of this study are available upon request. Requests for access to the data can be directed to Martina Pásková (martina.paskova@uhk.cz) and will be considered in accordance with the applicable data protection and privacy regulations. It is important to note that certain restrictions may apply to the availability of specific datasets due to confidentiality or ethical considerations. The researchers are committed to promoting transparency and reproducibility in research and will make every effort to provide access to the data in a timely and responsible manner.

## CRediT authorship contribution statement

**Martina Pásková:** Writing – review & editing, Writing – original draft, Supervision, Investigation, Data curation, Conceptualization. **Kamila Štekerová:** Writing – review & editing, Writing – original draft, Validation, Methodology, Investigation, Formal analysis, Conceptualization. **Marek Zanker:** Writing – review & editing, Writing – original draft, Validation, Project administration, Methodology, Investigation, Formal analysis, Data curation, Conceptualization. **Taiwo Temitope Lasisi:** Writing – review & editing, Writing – original draft. **Josef Zelenka:** Writing – review & editing, Writing – original draft, Supervision, Resources, Project administration, Investigation, Funding acquisition, Conceptualization.

## Declaration of Competing interest

The authors declare no conflict of interest. The funders had no role in the design of the study; in the collection, analyses, or interpretation of data; in the writing of the manuscript, or in the decision to publish the results.

## Figures and Tables

**Table 7:** Papers included in review

No.	Citation	Source	Location	Domain	SD artefacts	Platform	Scenarios
1	[80]	journal	Amtoudi Oasis	Sustainable tourism	CLD	Unspecified	Yes
2	[78]	journal	Taleqan County	Sustainable tourism	SFD	MapSys	Yes
3	[101]	conference	Chiang Mai city	Decision making	CLD, SFD	Vensim	No
4	[84]	journal	Nani Wartabone Bogani National Park	Destination management	CLD, SFD	Powersim	Yes
5	[104]	journal	Without location	Seaside tourism	CLD, SFD	Unspecified	No
6	[106]	journal	Kenting coastal zone	Seaside tourism	CLD, SFD	Stella	Yes
7	[107]	journal	Jiading Wetland	Wetland management	CLD, SFD	Vensim, Stella	Yes
8	[108]	journal	Mentougou district, Beijing	Destination management	SFD	Vensim	No
9	[83]	journal	Chiku coastal zone	Seaside tourism	CLD	Stella	Yes
10	[93]	journal	Global ocean	Waste management	SFD	Powersim	Yes
11	[130]	conference	Indonesia	Waste management	CLD	Vensim	No
12	[109]	journal	Qeensland	Renewable energy	SFD	Vensim	Yes
13	[96]	journal	Balearic Islands	Waste management	SFD	Vensim	Yes
14	[82]	journal	Canary Islands	Waste management	SFD	Vensim	Yes
15	[110]	journal	Maledives	Destination management	SFD	Vensim	Yes
16	[100]	conference	Tunisia	Tourism	CLD	Vensim	No
17	[127]	journal	General model, no location	Destination management	CLD	Unspecified	No
18	[111]	journal	European market	Sustainability	CLD, SFD	Vensim	Yes
19	[112]	journal	Taiwan	Agritourism	CLD, SFD	Vensim	Yes
20	[67]	journal	Not specified	Agritourism	CLD, SFD	Powersim	No
21	[86]	conference	Xidi and Hongcun World Heritage Villages in southern Anhui province	Destination management	CLD, SFD	Vensim	Yes
22	[94]	journal	Maledives	Waste management	SFD	Vensim	Yes
23	[90]	journal	Barents Sea and Northern Norwegian Sea region	Negative impacts	CLD, SFD	Stella	Yes
24	[113]	journal	Sepetiba Bay Watershed	Environmental Management	SFD	Stella	Yes
25	[75]	conference	Cijin	Sustainable tourism	CLD, SFD	Stella	Yes
26	[114]	journal	Xidi and Hongcun villages	Destination management	CLD, SFD	Vensim	Yes
27	[88]	journal	Beijing	Sustainable tourism	CLD, SFD	Vensim	No
28	[73]	journal	Dalian	Tourism in general	CLD, SFD	Vensim	Yes
29	[87]	journal	Not specified	Low carbon policy	CLD, SFD	Vensim	No
30	[79]	journal	Xingwen UNESCO Global Geopark	Sustainable tourism	CLD	Simulink	Yes
31	[98]	journal	Cat Ba Island	Sustainable tourism	CLD, SFD	Vensim, Stella	Yes
32	[95]	journal	Sagarmatha National Park and Buffer Zone	Waste management	CLD	Unspecified	Yes
33	[91]	journal	São Miguel	Low carbon policy	CLD	Vensim	Yes
34	[115]	conference	Not specified	Decision making	CLD, SFD	Powersim, Vensim	No
35	[126]	journal	Bali	Sustainable tourism	CLD, SFD	Powersim	Yes
36	[70]	conference	Cape Town	Tourism in general	SFD	Stella	No
37	[99]	journal	Hai Phong City	Sustainable tourism	CLD, archetypes	Vensim	No
38	[71]	journal	Surkarata City, Java	Tourism in general	CLD	Powersim	Yes
39	[102]	journal	Pieh marine park	Sustainability	CLD, SFD	Vensim	No
40	[129]	journal	Cat Ba Island	Negative impacts	CLD, archetypes	Vensim	No
41	[116]	journal	Bangkok	Flooding impacts	CLD, SFD	Vensim	Yes
42	[92]	journal	Galapagos Islands	tourism management	SFD	Unspecified	Yes
43	[97]	journal	Sicily	Destination management	CLD	Powersim	No
44	[69]	conference	Gucheng District of the City of Lijiang	Sustainable tourism	SFD	Stella	Yes
45	[117]	journal	Worldwide ocean	Accumlation of plastics	SFD	Venim	Yes
46	[128]	journal	Slovenia	Destination management	CLD	Unspecified	No
47	[119]	journal	Galapágos Islands	Island tourism	SFD	Vensim	Yes
48	[118]	journal	Stingray City Sandbar’ SCS, Cayman Islands	Destination management	CLD, SFD	Stella	Yes
49	[103]	conference	Amsterdam	Sustainable Tourism	CLD, SFD	Vensim, Stella	Yes
50	[120]	journal	Long Island Marine Stone Forest Park	Seaside tourism	CLD	Unspecified	No
51	[77]	journal	Rawa Danau forest, Indonesia	Destination management	CLD, SFD	Unspecified	Yes
52	[121]	conference	Sicily	Destination management	CLD, SFD	Powersim	Yes
53	[89]	journal	Karimunjawa National Park, Central Java	Sustainability	CLD, SFD	Vensim, Powersim	Yes
54	[74]	journal	Cijin, Kaohsiung	Sustainable tourism	CLD, SFD	Stella	Yes
55	[76]	journal	Ethiopia	Destination management	CLD, SFD	Vensim	No
56	[122]	journal	Not specified	Agriculture	SFD	Vensim	Yes
57	[68]	journal	Hinagdanan Cave	Sustainable tourism	CLD	Unspecified	Yes
58	[105]	journal	Dutch Wadden Sea region	Integrated coastal management	CLD, SFD	Vensim	No
59	Walsh and Mena (2014)	journal	Iconic national parks (in general	Negative impacts	SFD	Unspecified	No
60	[123]	journal	Tabanan Regency, Bali Province	Sustainable tourism	SFD	Powersim	Yes
61	[124]	journal	Shanghain	Waste manahement	CLD, SFD	Vensim	Yes
62	[81]	journal	South European island tourist economies	Sustainable tourism	CLD, SFD	Vensim	Yes
63	[125]	journal	Lanzhou City	Urban tourism	SFD	Vensim	Yes
64	[131]	journal	General model, no location	Planning	CLD	Vensim	No
65	[72]	journal	Shinduri coastal sand dune	Seaside tourism	SFD	Stella	Yes
66	[85]	journal	Tibet	Sustainable tourism	CLD, SFD	Vensim	Yes

## References

[1] Bondar A.I., Mashkov O.A., Zhukaskas S.V., Nygorodova S.A. (2019). Ecological threats, risks, and environmental terrorism: system definition. Ekologicni nauki.

[2] Pásková M., Zelenka J. (2019). How crucial is the social responsibility for tourism sustainability. Soc. Respons. J..

[3] Paiano A., Crovella T., Lagioia G. (2020). Managing sustainable practices in cruise tourism: the assessment of carbon footprint and waste of water and beverage packaging. Tourism Manage.

[4] Schuhmann P.W. (2013). Tourist perceptions of beach cleanliness in Barbados: implications for return visitation. Études Caribéennes.

[5] Dodds R., Holmes M.R. (2019). Beach tourists; what factors satisfy them and drive them to return. Ocean Coast. Manage..

[6] Ndong G.O., Therond O., Cousin I. (2020). Analysis of relationships between ecosystem services: a generic classification and review of the literature. Ecosyst. Serv..

[7] Nahuelhual L., Vergara X., Bozzeda F., Campos G., Subida M.D., Outeiro L., Villasante S., Fernández M. (2020). Exploring gaps in mapping marine ecosystem services: a benchmark analysis. Ocean Coast Manag..

[8] Shaltami O., Hamed N., Fares F., Errishi H., El Oshebi F., Maceda E. (October 2020). Virtual Proceedings of Conference on Environment and Health (VCEH).

[9] Khan S.A., Ali A. (2013). Baig, M.N. The linkage between agricultural practices and environmental degradation. Journal of Environmental Treatment Techniques.

[10] Landrigan P.J., Stegeman J.J., Fleming L.E., Allemand D., Anderson D.M., Backer L.C., Brucker-Davis F., Chevalier N., Corra L., Czerucka D., Bottein M.-Y.D., Demeneix B., Depledge M., Deheyn D.D., Dorman C.J., Fénichel P., Fisher S., Gaill F., Galgani F., Rampal P. (2020). Human health and ocean pollution. Annals of Global Health.

[11] Streimikiene D., Svagzdiene B., Jasinskas E., Simanavicius A. (2021). Sustainable tourism development and competitiveness: the systematic literature review. Sustain. Dev..

[12] Stern M.A., Flint L.E., Flint A.L., Knowles N., Wright S.A. (2020). The future of sediment transport and streamflow under a changing climate and the implications for long‐term resilience of the San Francisco bay‐delta. Water Resour. Res..

[13] Parra-Luna M., Martín-Pozo L., Hidalgo F., Zafra-Gómez A. (2020). Common sea urchin (Paracentrotus lividus) and sea cucumber of the genus Holothuria as bioindicators of pollution in the study of chemical contaminants in aquatic media. A revision. Ecol. Indicat..

[14] D'Angelo S., Meccariello R. (2021). Microplastics: a threat for male fertility. Int. J. Environ. Res. Publ. Health.

[15] Garcés-Ordóñez O., Espinosa Díaz L.F., Pereira Cardoso R., Costa Muniz M. (2020). The impact of tourism on marine litter pollution on Santa Marta beaches, Colombian Caribbean. Mar. Pollut. Bull..

[16] Petronella F., Comparelli R. (2021). Nanomaterials in photo (electro) catalysis. Catalysts.

[17] Mester T., Benkhard B., Vasvári M., Csorba P., Kiss E., Balla D., Fazekas I., Csépes E., Barkat A., Szabó G. (2023). Hydrochemical assessment of the kisköre reservoir (lake tisza) and the impacts of water quality on tourism development. Water.

[18] Folgado-Fernández J.A., Di-Clemente E., Hernández-Mogollón J.M. (2019). Campón-cerro, A.M. Water tourism: a new strategy for the sustainable management of water-based ecosystems and landscapes in extremadura (Spain). Land.

[19] Tyrrell T. (August 1992). Second Marine Debris Workshop.

[20] Iucn. Marine plastics https://www.iucn.org/resources/issues-brief/marine-plastic-pollution.

[21] Lebreton L., Zwet J., Damsteeg J.-W., Slat B., Andrady A., Reisser J. (2017). River plastic emissions to the world's oceans. Nat. Commun..

[22] Cole M., Lindeque P., Halsband C., Galloway T.S. (2011). Microplastics as contaminants in the marine environment: a review. Mar. Pollut. Bull..

[23] Wilson S.P., Verlis K.M. (2017). The ugly face of tourism: marine debris pollution linked to visitation in the southern Great Barrier Reef, Australia. Mar. Pollut. Bull..

[24] Wang F., Lai Z., Peng G., Luo L., Liu K., Huang X., Xu Y., Shen Q., Li D. (2021). Microplastic abundance and distribution in a Central Asian desert. Sci. Total Environ..

[25] Wang Ch, Zhao J., Xing B. (2021). Environmental source, fate, and toxicity of microplastics. J. Hazardous Mater..

[26] Roca E., Villares M. (2008). Public perceptions for evaluating beach quality in urban and semi-natural environments. Ocean Coast. Manage..

[27] Marin V., Palmisani F., Ivaldi R., Dursi R., Fabiano M. (2009). Users' perception analysis for sustainable beach management in Italy. Ocean Coast. Manage..

[28] Jang Y. Ch, Hong S., Lee J., Lee M.J., Shim W.J. (2014). Estimation of lost tourism revenue in Geoje Island from the 2011 marine debris pollution event in South Korea. Mar. Pollut. Bull..

[29] Schuhmann P.W., Bass B.E., Casey J.F., Gill D.A. (2016). Visitor preferences and willingness to pay for coastal attributes in Barbados. Ocean Coast Manag..

[30] Schuhmann P., Skeete R., Waite R., Bangwayo-Skeete P., Casey J., Oxenford H.A., Gill D.A. (2019). Coastal and marine quality and tourists' stated intention to return to Barbados. Water.

[31] Pásková M. (2012). Environmentalistika cestovního ruchu [tourism environmentalism]. Czech J. Tourism.

[32] Priporas C.V., Stylos N., Rahimi R., Vedanthachari L.N. (2017). Unraveling the diverse nature of service quality in a sharing economy: a social exchange theory perspective of Airbnb accommodation. Int. J. Contemp. Hosp. M..

[33] Chen S., Raab C. (2012). Predicting resident intentions to support community tourism: toward an integration of two theories. J. Hosp. Mark. M..

[34] Robina-Ramírez R., Sánchez-Oro M., Cabezas-Hernández M., Calleja-Aldana M. (2020). Host and guest social exchange in developing tourist sites: the case of the International Tagus Natural Park. Sustainability.

[35] Aljerf L. (2015). Change theories drift conventional tourism into ecotourism. Acta Technica Corviniensis – Bull. Engin..

[36] Grelaud M., Ziveri P. (2020). The generation of marine litter in Mediterranean island beaches as an effect of tourism and its mitigation. Sci. Rep..

[37] Saint Akadiri S., Lasisi T.T., Uzuner G., Akadiri A.C. (2019). Examining the impact of globalization in the environmental Kuznets curve hypothesis: the case of tourist destination states. Environ. Sci. Pollut. R..

[38] Eluwole K.K., Bekun F.V., Lasisi T.T. (2022). Fresh insights into tourism-led economic growth nexus: a systematic literature network analysis approach. Asia Pac. J. Tourism Res..

[39] Nematollahi M.J., Keshavarzi B., Moore F., Esmaeili H.R., Nasrollahzadeh Saravi H., Sorooshian A. (2021). Microplastic fibers in the gut of highly consumed fish species from the southern Caspian Sea. Mar. Pollut. Bull..

[40] Bonanno G., Orlando-Bonaca M. (2018). Ten inconvenient questions about plastics in the sea. Environ. Sci. Policy.

[41] Forschungsverbund Berlin, An underestimated threat: Land-based pollution with microplastics. ScienceDaily, Available online: https://www.sciencedaily.com/releases/2018/02/180205125728.htm (accessed on 5 June 2021).

[42] Clunies-Ross (2019).

[43] Lebreton L., Andrady A. (2019). Future scenarios of global plastic waste generation and disposal. Palgrave Commun.

[44] Wongprapinkul B., Vassanadumrongdee S. (2022). A systems thinking approach towards single-use plastics reduction in food delivery business in Thailand. Sustainability.

[45] Rosian Charity. Tourism and plastic pollution., Available online: https://rosian.org/posts/tourism-and-plastic-pollution, (accessed on 21 June 2021).

[46] Chen P., Kong X. (2021). Tourism-led commodification of place and rural transformation development: a case study of xixinan village, huangshan, China. Land.

[47] Ashworth G., Page S.J. (2011). Urban tourism research: recent progress and current paradoxes. Tourism Manag..

[48] Garrod B., Wornell R., Youell R. (2006). Re-conceptualising rural resources as countryside capital: the case of rural tourism. J. Rural Stud..

[49] Saxena G., Clark G.L., Oliver T., Ilbery B. (2007). Conceptualizing integrated rural tourism. Tourism Geogr..

[50] Sánchez-Quiles D., Tovar-Sánchez A. (2015). Are sunscreens a new environmental risk associated with coastal tourism?. Environ. Int..

[51] Light D., Creţan R., Voiculescu S., Jucu I.S. (2020). Introduction: changing tourism in the cities of post-communist central and eastern europe. Journal of Balkan and Near Eastern Studies.

[52] Mikhailenko A.V., Ruban D.A., Ermolaev V.A., Loon A. J. van (2020). Cadmium pollution in the tourism environment: a literature review. Geosciences.

[53] Ouyang T., Zhu Z., Kuang Y. (2006). Assessing impact of urbanization on river water quality in the Pearl River Delta economic zone, China. Environ. Monit. Assess..

[54] Azar A.T. (2012). System dynamics is a useful technique for complex systems. Int. J. Ind. Syst. Eng..

[55] Zarghami S.A., Gunawan I., Schultmann F. (2018). System dynamics modelling process in water sector: a review of research literature. Syst. Res. Behav. Sci..

[56] Mousavi S.H., Kavianpour M.R., Alcaraz J.L.G., Yamini O.A. (2023). System dynamics modeling for effective strategies in water pollution control: insights and applications. Appl. Sci..

[57] Nagendrababu V., Dilokthornsakul P., Jinatongthai P., Veettil S.K., Pulikkotil S.J., Duncan H.F., Dummer P.M.H. (2020). Glossary for systematic reviews and meta‐analyses. Int. Endod. J..

[58] Tricco A.C., Lillie E., Zarin W., O'Brien K.K., Colquhoun H., Levac D., Moher D., Peters M.D.J., Horsley T., Weeks L., Hempel S., Akl E.A., Chang C., McGowan J., Stewart L., Hartling L., Aldcroft A., Wilson M.G., Garritty C., Straus S.E. (2018). PRISMA extension for scoping reviews (PRISMA-ScR): checklist and explanation. Ann. Intern. Med..

[59] Page M.J., Moher D., Bossuyt P.M., Boutron I., Hoffmann T.C., Mulrow C.D., Shamseer L., Tetzlaff J.M., Akl E.A., Brennan S.E., Chou R., Glanville J., Grimshaw J.M., Hróbjartsson A., Lalu M.M., Li T., Loder E.W., Mayo-Wilson E., McDonald S., McKenzie J.E. (2021). PRISMA 2020 explanation and elaboration: updated guidance and exemplars for reporting systematic reviews. BMJ.

[60] Moher D., Shamseer L., Clarke M., Ghersi D., Liberati A., Petticrew M., Shekelle P., Stewart L.A. (2015). Preferred reporting items for systematic review and meta-analysis protocols (PRISMA-P) 2015 statement. Syst. Rev..

[61] Peters M.D., Godfrey C., McInerney P., Munn Z., Tricco A.C., Khalil H. (2020). JBI Manual for Evidence Synthesis1.

[62] Sarkis-Onofre R., Catalá-López F., Aromataris E., Lockwood C. (2021). How to properly use the PRISMA Statement. Syst. Rev..

[63] Davahli M.R., Karwowski W., Taiar R. (2020). A system dynamics simulation applied to healthcare: a systematic review. Int. J. Env. Res. Pub. He..

[64] Fontoura W., Ribeiro G. (2021). System dynamics for sustainable transportation policies: a systematic literature review. urbe. Revista Brasileira de Gestão Urbana.

[65] Liu M., Le Y., Hu Y., Xia B., Skitmore M., Gao X. (2019). System dynamics modeling for construction management research: critical review and future trends. J. Civ. Eng. Manag..

[66] Zanker M., Štekerová K. (2020). Proceedings of the International Scientific Conference Hradec Economic Days 2020.

[67] Jere Jakulin T. (2016). System dynamics models as decision-making tools in agritourism. Agricultura.

[68] Uy J.A., Escalante N.L.S., Tonggol H.M.M., Radomes A.A. (2018). An empirical multidimensional analysis on sustainable tourism: the dynamics of carrying capacity. Int. J. Tourism Policy.

[69] Ran W. (2015). Proceedings of 30th International Conference of the System Dynamics Society.

[70] Mona S. (2018). Proceedings of the International Conference on Industrial Engineering and Operations Management.

[71] Novani S., Azis Y., Aprianingsih A., Aru A.P., Putro U.S. (2019). Collaboration improvement among batik tourism stakeholders of Surakarta City: a value co-creation process with soft system dynamics methodology. Intern. J. Business Innov. Res..

[72] You S., Kim M., Lee J., Chon J. (2018). Coastal landscape planning for improving the value of ecosystem services in coastal areas: using system dynamics model. Environ. Pollut..

[73] Lu X., Yao S., Fu G., Lv X., Mao Y. (2019). Dynamic simulation test of a model of ecological system security for a coastal tourist city. J. Destin. Mark. Manage..

[74] Tan W.-J., Yang C.-F., Château P.-A., Lee M.-T., Chang Y.-C. (2018). Integrated coastal-zone management for sustainable tourism using a decision support system based on system dynamics: a case study of Cijin, Kaohsiung, Taiwan. Ocean Coast. Manage..

[75] Lee M.T., Lin T.F. (2014). Proceedings of 2014 International Symposium on Computer, Consumer and Control (IS3C).

[76] Tegegne W.A., Moyle B.D., Becken S. (2018). A qualitative system dynamics approach to understanding destination image. J. Destin. Mark. Manage..

[77] Sjaifuddin S. (2020). Sustainable management of freshwater swamp forest as an ecotourism destination in Indonesia: a system dynamics modeling. Entrepreneurship and Sustainability Issues.

[78] Aliani H., Kafaky S.B., Monavari S.M., Dourani K. (2018). Modeling and prediction of future ecotourism conditions applying system dynamics. Environ. Monit. Assess..

[79] Luo Y., Mou Y., Wang Z., Su Z., Qin Y. (2020). Scenario-based planning for a dynamic tourism system with carbon footprint analysis: a case study of Xingwen Global Geopark, China. J. Clean. Prod..

[80] Alcalá F.J., Martínez-Valderrama J., Robles-Marín P., Guerrera F., Martín-Martín M., Raffaelli G., León J.T., Asebriy L. (2015). A hydrological-economic model for sustainable groundwater use in sparse-data drylands: application to the Amtoudi Oasis in southern Morocco, northern Sahara. Sci. Total Environ..

[81] Xing Y., Dangerfield B. (2011). Modelling the sustainability of mass tourism in island tourist economies. J. Oper. Res. Soc..

[82] Estay-Ossandon C., Mena-Nieto A., Harsch N. (2018). Using fuzzy TOPSIS-based scenario analysis to improve municipal solid waste planning and forecasting: a case study of Canary archipelago (1999–2030). J. Clean. Prod..

[83] Chiu C.-C., Château P.-A., Lin H.-J., Chang Y.-C. (2019). Modeling the impacts of coastal land use changes on regional carbon balance in the Chiku coastal zone, Taiwan. Land Use Pol..

[84] Bempah I. (2018). Dynamics analysis model of nature tourism system development in bogani nani wartabone national park of gorontalo province. Jurnal Manajemen.

[85] Zhang J., Ji M., Zhang Y. (2015). Tourism sustainability in Tibet - forward planning using a systems approach. Ecol. Indicat..

[86] Jiang J., Li J., Xu H. (July 2010). 28th International Conference of the System Dynamics Society 2010.

[87] Luo Y., Jin M., Ren P., Liao Z., Zhu Z. (2014). Simulation and prediction of decarbonated development in tourist attractions associated with low-carbon economy. Sustainability.

[88] Liao Z., Jin M., Ren P., Luo Y. (2014). Research on scenic spot's sustainable development based on a SD model: a case study of the Jiuzhai Valley. Sustainability.

[89] Susanty A., Puspitasari N.B., Saptadi S., Siregar S.D. (2020). Using system dynamics approach to build policy scenario for reducing CO_2_ emission resulted from tourism travel to Karimunjawa. Kybernetes.

[90] Koenigstein S., Ruth M., Gößling-Reisemann S. (2016). Stakeholder-informed ecosystem modeling of ocean warming and acidification impacts in the Barents Sea Region. Frontiers in Marine Sci.

[91] Matthew G., Nuttall W.J., Mestel B., Dooley L.S. (2017). A dynamic simulation of low-carbon policy influences on endogenous electricity demand in an isolated island system. Energ. Policy.

[92] Pizzitutti F., Walsh S.J., Rindfuss R.R., Gunter R., Quiroga D., Tippett R., Mena C.F. (2017). Scenario planning for tourism management: a participatory and system dynamics model applied to the Galapagos Islands of Ecuador. J. Sustain. Tour..

[93] Cordier M., Uehara T. (2019). Will innovation solve the global plastic contamination: how much innovation is needed for that?. Sci. Total Environ..

[94] Kapmeier F., Gonçalves P. (2018). Wasted paradise? Policies for Small Island States to manage tourism-driven growth while controlling waste generation: the case of the Maldives. Syst. Dynam. Rev..

[95] Manfredi E.C., Flury B., Viviano G. (2010). Solid waste and water quality management models for sagarmatha national park and buffer zone, Nepal implementation of a participatory modeling framework. Mt. Res. Dev..

[96] Estay-Ossandon C., Mena-Nieto A. (2018). Modelling the driving forces of the municipal solid waste generation in touristic islands. A case study of the Balearic Islands (2000–2030). Waste Manage. (Tucson, Ariz.).

[97] Provenzano D. (2015). A dynamic analysis of tourism determinants in sicily. Tourism Econ..

[98] Mai T., Smith C. (2018). Scenario-based planning for tourism development using system dynamic modelling: a case study of Cat Ba Island, Vietnam. Tourism Manage..

[99] Nguyen N.C., Bosch O.J.H. (2013). A systems thinking approach to identify leverage points for sustainability: a case study in the Cat Ba biosphere Reserve, Vietnam: using systems thinking to identify leverage points for sustainability. Syst. Res. Behav. Sci..

[100] Halioui S., Schmidt M. (2016). Proceedings of WEI Conference at.

[101] Asasuppakit P., Thiengburanathum P. (2014). Proceedings of the 2014 Asia-Pacific System Dynamics Conference.

[102] Nugroho S., Uehara T., Herwangi Y. (2019). Interpreting daly's sustainability criteria for assessing the sustainability of marine protected areas: a system dynamics approach. Sustainability.

[103] Sharma M., Sehrawat R. (2019). Proceedings of 2019 International Symposium on Advanced Electrical and Communication Technologies.

[104] Brennan C., Ashley M., Molloy O. (2019). A system dynamics approach to increasing ocean literacy. Frontiers Marine Sci.

[105] Vugteveen P., Rouwette E., Stouten H., van Katwijk M.M., Hanssen L. (2015). Developing social-ecological system indicators using group model building. Ocean Coast Manage.

[106] Chang Y.C., Hong F.W., Lee M.T. (2008). A system dynamic based DSS for sustainable coral reef management in Kenting coastal zone, Taiwan. Ecol. Model..

[107] Chen H., Chang Y.-C., Chen K.-C. (2014). Integrated wetland management: an analysis with group model building based on system dynamics model. J. Environ. Manage..

[108] Cheng L., Sun H., Zhang Y., Zhen S. (2019). Spatial structure optimization of mountainous abandoned mine land reuse based on system dynamics model and CLUE-S model. Internat. J. of Coal Sci. Technol..

[109] Dhirasasna N., Sahin O. (2021). A system dynamics model for renewable energy technology adoption of the hotel sector, Renew. Energ..

[110] Gu Y., Onggo B.S., Kunc M.H., Bayer S. (2021). Small Island Developing States (SIDS) COVID-19 post-pandemic tourism recovery: a system dynamics approach. Curr. Issues Tour..

[111] Horvat D., Wydra S., Lerch C.M. (2018). Modelling and simulating the dynamics of the European demand for bio-based plastics. Int J Simul Model.

[112] Hsiao T.Y., Hsu Y.Y. (2014). Modeling different scenarios for forecasting human resources requirements in taiwan's recreational farms. Internat. J. Bus. Admin..

[113] Leal Neto A.D.C., Legey L.F.L., González-Araya M.C., Jablonski S. (2006). A system dynamics model for the environmental management of the Sepetiba Bay Watershed, Brazil. Environ. Manage..

[114] Li J., Zhang W., Xu H., Jiang J. (2015). Dynamic competition and cooperation of road infrastructure investment of multiple tourism destinations: a case study of xidi and hongcun world cultural heritage. Discrete Dyn. Nat. Soc..

[115] McGrath G.M. (2010). Proceedings of 43rd Hawaii International Conference on System Sciences.

[116] Phonphoton N., Pharino C. (2019). A system dynamics modeling to evaluate flooding impacts on municipal solid waste management services. Waste Manage. (Tucson, Ariz.).

[117] Rellán G.A., Vázquez Ares D., Vázquez Brea C., Francisco López A., Bello Bugallo P.M. (2023). Sources, sinks and transformations of plastics in our oceans: review, management strategies and modelling. Sci. Total Environ..

[118] Semeniuk C.A.D., Haider W., Cooper A., Rothley K.D. (2010). A linked model of animal ecology and human behavior for the management of wildlife tourism. Ecol. Model..

[119] Sampedro C., Pizzitutti F., Quiroga D., Walsh S.J., Mena C.F. (2018). Food supply system dynamics in the Galapagos Islands: agriculture, livestock and imports. Renew. Agr. Food Syst..

[120] Shen Y. (2019). System dynamics model of long island marine stone forest park based on recreational opportunity spectrum. J. Coastal Res..

[121] Soufivand M., Alessi M., Bivona E. (2013). Proceedings of the 31th International Conference of the System Dynamics Society.

[122] Tita V., Mocuta D.-N., Turek-Rahoveanu A., Popescu D.A., Bold N. (2019). Integrated plastic management system within an agricultural enterprise analysis of actual context, system model and simulation. Mater. Plast..

[123] Widhianthini W. (2017). A dynamic model for sustainable tourism village planning based on local institutions. J. Region. City Plann..

[124] Xiao S., Dong H., Geng Y., Tian X., Liu C., Li H. (2020). Policy impacts on Municipal Solid Waste management in Shanghai: A system dynamics model analysis. J. Clean. Prod..

[125] Yang X., Jia Y., Zhang D., Zhang X., Zhang H., Hou Y. (2020). Research on the anti- interference capability of the tourism environment system for the core stakeholders of semi-arid valley-type cities: analysis based on the multi-scenario and time series diversity perspectives. Environ. Sci. Pollut. R..

[126] McGrath G.M., Law A., DeLacy T. (2015). Green economy planning in tourism destinations: an integrated, multi-method decision support aid. J. Dev. Areas.

[127] Haraldsson H., Ólafsdóttir R. (2018). Evolution of tourism in natural destinations and dynamic sustainable thresholds over time. Sustainability.

[128] Ropret M., Jere Jakulin T., Likar B. (2014). The systems approach to the improvement of innovation in Slovenian tourism. Kybernetes.

[129] Phan T.D., Nguyen N.C., Bosch O.J.H., Nguyen T.V., Le T.T., Tran H.T. (2016). A systemic approach to understand the conservation status and viability of the critically endangered Cat Ba langur: conservation status of the critically endangered Cat Ba langur. Syst. Res. Behav. Sci..

[130] Destyanto A.R., Kirana P.S., Ardi R. (2019). Proceedings of the 2019 5th International Conference on Industrial and Business Engineering - ICIBE 2019.

[131] Yin J., Zheng X., Tsaur R.-C. (2019). Occurrence mechanism and coping paths of accidents of highly aggregated tourist crowds based on system dynamics. PLoS One.

[132] Nozari H., Moradi P., Godarzi E. (2021). Simulation and optimization of control system operation and surface water allocation based on system dynamics modeling. J. Hydroinf..

[133] Lagarda-Leyva E.A. (2021). System dynamics and lean approach: development of a technological solution in a regional product packaging company. Appl. Sci..

[134] Boudjana S., Tadjine M. (2020). On cascaded loop-shaping/hybrid mode control design of three-cell inverter. Electrical Engineering.

[135] Yan Z., Zeng J., Zhang W., Feng P., Li Y., Yao C., Ma G. (2021). Dynamic simulation of vibration characteristics and ride quality of superconducting EDS train considering body with flexibility. IEEE Trans. Appl. Supercond..

[136] Walsh S., Carter R., Lieske S., Quiroga D., Mena C. (1999). Examining threats to iconic national parks through wang, N. Rethinking authenticity in tourism experience. Ann. Tourism Res..

[137] Iuras I., Raiter P., Korobeinykova Y., Poberezhna L. (2020). Methodology of actors analysis and modeling of the amounts of solid municipal waste generation within tourist destinations. Ecol. Quest..

[138] Arini D.U., Mardianta A.V. (2022). Waste water management in supporting sustainable tourism in girsang sipangan bolon District. International Journal of Architecture and Urbanism.

[139] Ma C.-Y., Huang Y., Kao C. (2018). Development of optimal management strategies for the interception system using river water quality modeling. Matec Web of Conferences.

[140] Stylidis D., Biran A., Sit K., Szivás E. (2014). Residents' support for tourism development: the role of residents' place image and perceived tourism impacts. Tourism Manag..

[141] McGehee N.G., Andereck K. (2004). Factors predicting rural residents' support of tourism. J. Trav. Res..

[142] Afsar B., Umrani W.A. (2019). Corporate social responsibility and pro‐environmental behavior at workplace: the role of moral reflectiveness, coworker advocacy, and environmental commitment. Corp. Soc. Responsib. Environ. Manag..

[143] Buckley R. (2012). Sustainable tourism: research and reality. Ann. Tourism Res..

[144] Li X. (2022). Green innovation behavior toward sustainable tourism development: a dual mediation model. Front. Psychol..

[145] Munfarida I., Nilandita W., Auvaria S.W. (2022). An environmental impact assessment of geothermal tourism: a case study of awit sinar alam darajat, garut-Indonesia. IOP Conf. Ser. Earth Environ. Sci..

[146] Deffinika I., Regina Heni Prastiwi M., Arinta D. (2022). Application of cultural tourism using community-based tourism in the kayutangan heritage area of malang city. KnE Social Sciences.

[147] Tian C., Peng J.J., Zhang W.Y., Zhang S., Wang J.Q. (2020). Tourism environmental impact assessment based on improved ahp and picture fuzzy promethee ii methods. Technol. Econ. Dev. Econ..

[148] Baum T., Kralj A., Robinson R.N.S., Solnet D.J. (2016). Tourism workforce research: a review, taxonomy, and agenda. Ann. Tourism Res..

[149] Page S.J., Essex S., Causevic S. (2014). Tourist attitudes towards water use in the developing world: a comparative analysis. Tourism Manag. Perspect..

[150] Băndoi A., Jianu E., Enescu M., Axinte G., Tudor S., Firoiu D. (2020). The Relationship between the development of tourism, quality of life, and sustainable performance in EU countries. Sustainability.

[151] Haddouche H., Salomone C. (2018). Generation Z and the tourist experience: tourist stories and use of social networks. J. Tourism Futur..

[152] Hadjikakou M., Chenoweth J., Miller G. (2013). Estimating the direct and indirect water use of tourism in the eastern Mediterranean. J. Environ. Manag..

[153] Zolfani S.H., Sedaghat M., Maknoon R., Zavadskas E.K. (2015). Sustainable tourism: a comprehensive literature review on frameworks and applications. Economic Research-Ekonomska Istraživanja.

[154] Mai T., Smith C. (2015). Addressing the threats to tourism sustainability using systems thinking: a case study of Cat Ba Island, Vietnam. J. Sustain. Tourism.

[155] Zulpikar F., Handayani T. (2021). Life form, diversity, and spatial distribution of macroalgae in komodo national park waters, east nusa tenggara. IOP Conf. Ser. Earth Environ. Sci..

[156] Martins A.M., Cró S. (2021). The impact of tourism on solid waste generation and management cost in madeira island for the period 1996–2018. Sustainability.

[157] Mateu-Sbert J., Ricci-Cabello I., Villalonga-Olives E., Cabeza-Irigoyen E. (2013). The impact of tourism on municipal solid waste generation: the case of Menorca Island (Spain). Waste Management.

[158] Munfarida I., Nilandita W., Auvaria S.W. (2022). An environmental impact assessment of geothermal tourism: a case study of Awit Sinar Alam Darajat, Garut-Indonesia. IOP Conf. Ser. Earth Environ. Sci..

[159] Bhammar H., Li W., Molina C.M.M., Hickey V., Pendry J., Narain U. (2021). Framework for sustainable recovery of tourism in protected areas. Sustainability.

[160] Khan M.R., Khan H.U.R., Lim C.K., Tan K.L., Ahmed M.F. (2021). Sustainable tourism policy, destination management and sustainable tourism development: a moderated-mediation model. Sustainability.

[161] Tiwari S., Rosak-Szyrocka J., Żywiołek J. (2022). Internet of things as a sustainable energy management solution at tourism destinations in India. Energies.

[162] Sun Q., Liu Z. (2020). Impact of tourism activities on water pollution in the West Lake basin (hangzhou, China). Open Geosci..

[163] Ravinashree A., Sivapragasam C., Vasudevan M. (2022). Developmental strategies for a water quality assessment model with limited datasets – a case study from river bhavani, India. IOP Conf. Ser. Earth Environ. Sci..

[164] Tosun C. (2000). Limits to community participation in the tourism development process in developing countries. Tourism Manag..

[165] Ștefănică M., Sandu C.B., Butnaru G.I., Haller A.-P. (2021). The nexus between tourism activities and environmental degradation: Romanian tourists' opinions. Sustainability.

[166] Kabera C., Tushabe E. (2021). Environmental conservation, A factor for promoting tourism industry in Rwanda: a case study of rubavu District. East African Journal of Environment and Natural Resources.

[167] Wang M.-C., Wang C.-S. (2018). Tourism, the environment, and energy policies. Tourism Econ..

[168] Lee T.H., Jan F.-H. (2015). The influence of recreation experience and environmental attitude on the environmentally responsible behavior of community-based tourists in Taiwan. J. Sustain. Tourism.

[169] Wang J., Dai J., Dewancker B.J., Gao W., Liu Z., Zhou Y. (2022). Impact of situational environmental education on tourist behavior—a case study of water culture ecological park in China. Int. J. Environ. Res. Publ. Health.

[170] Su L., Swanson S.R. (2017). The effect of destination social responsibility on tourist environmentally responsible behavior: compared analysis of first-time and repeat tourists. Tourism Manag..

[171] Grilli G., Tyllianakis E., Luisetti T., Ferrini S., Turner R.K. (2021). Prospective tourist preferences for sustainable tourism development in Small Island Developing States. Tourism Manag..

[172] Klöck C., Nunn P.D. (2019). Adaptation to climate change in small island developing states: a systematic literature review of academic research. J. Environ. Dev..

[173] Nunn P., Kumar R. (2018). Understanding climate-human interactions in small island developing states (SIDS). International Journal of Climate Change Strategies and Management.

[174] Loureiro S.M.C., Guerreiro J., Han H. (2022). Past, present, and future of pro-environmental behavior in tourism and hospitality: a text-mining approach. J. Sustain. Tourism.

[175] Pahlevan-Sharif S., Mura P., Wijesinghe S.N.R. (2019). A systematic review of systematic reviews in tourism. J. Hosp. Tourism Manage..

[176] Yung R., Khoo-Lattimore C. (2019). New realities: a systematic literature review on virtual reality and augmented reality in tourism research. Curr. Issues Tour..

[177] Naeem N., Rana I.A. (2020). Tourism and disasters: a systematic review from 2010–2019. J. Extre. Events.

[178] Estevão C., Costa C. (2020). Natural disaster management in tourist destinations: a systematic literature review. Eur. J. Tour. Res..

[179] Lama S., Pradhan S. (2020). https://aisel.aisnet.org/acis2020/17.

[180] Kanga Idé S., Seydou Niandou M.A., Naimi M., Chikhaoui M., Schimmel K., Luster-Teasley S., Sheikh N. (2019). A systematic review and meta-analysis of water quality indices. J. Agric. Sci. Technol..

